# From Detection to Prediction: A Systematic Review of Intelligent Fault Detection in Industrial Conveyor Belt Systems

**DOI:** 10.3390/s26216741

**Published:** 2026-10-24

**Authors:** Mounia Achouch, Rizck Dhouib, Romain Baudouin, Kong-Solyvan Ear, Christopher Mitride, Eric Sénéchal

**Affiliations:** 1 Centre de Recherche et D’innovation en Intelligence Énergétique CR2ie, 175 Rue de la Vérendrye, Sept-Îles, QC G4R 5B7, Canada; 2 Institut Technologique de Maintenance Industrielle ITMI, 175 Rue de la Vérendrye, Sept-Îles, QC G4R 5B7, Canada; rizck.dhouib@itmi.ca (R.D.); romain.baudouin@cegepsi.ca (R.B.); solyvan.ear.kong@itmi.ca (K.-S.E.); eric.senechal@itmi.ca (E.S.)

**Keywords:** conveyor systems, predictive maintenance, anomaly detection, multi-sensor fusion, deep learning, machine learning, computer vision, edge computing, industrial IoT

## Abstract

**Highlights:**

**What are the main findings?**

- Intelligent conveyor belt fault detection increasingly relies on deep learning, with CNN-based vision models becoming the dominant analytical approach.
- Effective fault detection depends on matching the sensing and analytical method to the specific fault mechanism and deployment conditions.
- Existing diagnostic approaches remain concentrated on fault detection rather than prediction; only 2.3% of the studies estimate remaining useful life.

**What are the implications of the main findings?**

- Development of prognosis-oriented and physics-informed modeling frameworks may improve the predictive value and transferability of future diagnostic systems.
- Integration of principled multi-sensor fusion with calibrated, explainable inference represents a promising direction toward genuinely predictive and trustworthy conveyor belt monitoring.

**Abstract:**

Industrial conveyor belt systems are critical infrastructure where unexpected failures cause major downtime, safety hazards, and economic losses, motivating growing research on AI-based fault detection. However, previous reviews have either focused on a single sensing modality, particularly computer vision, or considered conveyor systems within broader mining applications, without jointly assessing methodological quality, cross-modal sensing, deployment readiness, and the technical capabilities required for prognosis. Following PRISMA 2020 guidelines, this review analyzes 131 empirical studies on intelligent fault detection in industrial conveyor belt systems published between 2010 and 2026, with particular attention to sensing technologies, AI-based analytical methods, and deployment feasibility. The analysis considers the main fault types reported in the literature and the sensors and algorithms used for their detection. Deep learning was the dominant analytical approach (55.0% of studies), with CNN-based architectures such as YOLO, ResNet, and U-Net widely used for vision-based applications, while LSTM and GRU models were more commonly applied to acoustic and vibration signals. Vision/camera sensing accounted for 40.5% of studies, and mining represented 74% of applications. Despite substantial progress in automated fault detection, most studies remain focused on identifying existing faults rather than anticipating future degradation. Remaining useful life was estimated in only three studies (2.3%) and degradation trends in seven (5.3%); physics-informed machine learning and cross-site domain adaptation were absent, and principled multi-sensor fusion, uncertainty quantification, explainability, and digital-twin integration each appeared in no more than six studies. Predictive uncertainty was quantified in only one study (0.8%), and its calibration was not demonstrated. Future advances should therefore place greater emphasis on prognosis, cross-site generalization, uncertainty-aware inference, explainable models, and principled sensor fusion to support more reliable and transferable predictive maintenance systems.

## 1. Introduction

Conveyor systems are essential for many industries, such as mining, ports, and manufacturing, to transport materials and products safely and efficiently [1,2,3]. For instance, in mining, conveyor belts support continuous bulk material handling in harsh environments, motivating research on early damage/anomaly detection and safety-oriented inspection [4,5,6]. Moreover, to improve productivity and minimize the risk of interruptions in ports and industrial facilities, the automated monitoring of conveyor belt and idler conditions has been proposed [7,8,9,10]. However, if a conveyor system fails, it has significant consequences, as a minor fault may disrupt the entire system, leading to unexpected downtime and financial losses [1,3,11,12]. This is especially critical in continuous flow operations, where even short interruptions can generate downstream issues across production and supply chains [1,13].

Traditional maintenance of conveyor systems has mainly focused on manual or periodic inspection to identify any signs of wear or failure in components such as belts and idlers [6,14,15,16]. Although these approaches are simple, they are prone to subjectivity and may miss early-stage defects, particularly under challenging field conditions [4,5,17,18]. Many industries use schedule-driven interventions for different operations to avoid failure in a conveyor system, which can reduce catastrophic events but may increase unnecessary maintenance actions and operational costs [3,11]. Conventional sensing has been widely used to improve fault detection: vibration monitoring has been applied to characterize belt/roller–idler dynamics and diagnose rotating components [19,20,21], while thermal/infrared approaches detect abnormal heating patterns associated with degradation, mis-rotation, or localized resistance [16,22,23]. Increasingly, these approaches are being augmented or replaced by intelligent, data-driven systems to improve diagnostic accuracy and automation [1,13,24].

Under Industry 4.0, research has increasingly focused on intelligent conveyor maintenance using data-driven approaches [1,24,25]. Anomaly and fault detection in conveyor systems are becoming earlier, faster, and increasingly automated by integrating artificial intelligence (AI), Industrial Internet of Things (IIoT) connectivity, and edge/cloud computing as modern solutions [24,26,27]. With the emergence of smart sensing, operational parameters of conveyor systems are being continuously collected, thereby facilitating the acquisition of high-quality operational data (e.g., vibration/thermal/telemetry) [21,23,25]. These data can be processed locally (on-device/edge) or via connected architectures using machine learning and deep learning models for anomaly detection and predictive diagnostics [24,27,28]. In parallel, digital-twin approaches have been proposed to create a virtual representation of the conveyor system for testing, prediction, and maintenance strategy evaluation in a low-risk setting [3,29,30]. These intelligent systems aim to improve productivity, reduce downtime and costs, and support real-time decision-making and longer-term maintenance planning [1,3,11]. Related research on milling-machine fault diagnosis using CWT-based image augmentation and optimized deep learning [31] and uncertainty-calibrated anomaly monitoring of wind-turbine SCADA data under distribution shift [32] provides complementary methodological perspectives for intelligent industrial condition monitoring.

Although significant improvements have been made in the detection of anomalies that occur in conveyor systems, existing maintenance practices remain insufficient for many modern industrial requirements. This is because manual inspection processes are time-consuming, irregular, and difficult to scale [17,22,33,34]. Additionally, the efficiency of intelligent inspection might be reduced under harsh conditions, such as those with poor lighting, noise, and clutter, which often corrupt the signal or affect the image quality [4,6,18]. Also, models developed in one setup may not be adapted to other sites or belt types, emphasizing the issue of transferability [24,25,35]. Real-time processing can also be constrained by edge hardware limits (e.g., compute, memory, energy/latency) [24,25]. Finally, limited labeled fault data and the need for standardized datasets remain persistent barriers for robust training and validation [5,34,36,37]. These challenges reinforce the need for intelligent, flexible maintenance systems that remain robust in dynamic industrial conditions.

Several reviews have examined intelligent conveyor monitoring, but their scope remains relatively narrow or addresses conveyor systems only as part of a broader asset class. For instance, Rzeszowska et al. [38] reviewed sensing and signal-based approaches for conveyor belt damage detection but did not include a formal assessment of methodological quality or deployment characteristics. Rios-Colque et al. [39] focused specifically on computer vision-based techniques for conveyor belt monitoring and identified prognosis, generalization, and industrial integration as important future directions, but acoustic, vibration, thermal, and magnetic modalities were outside their review scope. Rojas et al. [40] examined AI-based predictive maintenance across several mining assets, with limited focus on conveyor systems. These reviews establish the importance of moving beyond fault detection, but they do not quantify whether the conveyor-monitoring literature currently provides the sensing, validation, and deployment capabilities required for that transition. The present review addresses this gap by jointly evaluating multiple sensing modalities, AI methods, methodological quality, external validation, reproducibility, deployment architecture, cost reporting, and advanced predictive capabilities, including RUL estimation, physics-informed modeling, principled sensor fusion, domain adaptation, calibrated uncertainty, explainability, and digital-twin integration. This broader assessment is used to evaluate the readiness of current conveyor-monitoring research to progress from fault detection toward reliable predictive maintenance in Industry 4.0 environments.

This paper is organized as follows: Section 2 describes the review design, search strategy, eligibility criteria, protocol status, and methodological appraisal. Section 3 gives a quantitative overview of the included studies, including their methodological quality, validation settings, deployment architecture, and cost reporting. Section 4 analyzes fault types, sensing modalities, and modeling approaches. Section 5 discusses the reporting gaps and the scientific and technical gaps that limit the transition from detection to prediction, and Section 6 concludes the review.

## 2. Materials and Methods

### 2.1. Review Design and Reporting Framework

We conducted this review in accordance with the PRISMA 2020 guidelines [41] to support transparent and structured reporting of the search, screening, and synthesis process. This review addresses three main research questions: (Q1) What are the most studied components of the conveyor system and types of anomalies in smart maintenance research, and what types of sensors are used for this purpose? (Q2) What are the most promising artificial intelligence or machine learning techniques for achieving good trade-offs between detection accuracy, speed, and deployment feasibility (edge vs. cloud)? (Q3) What are the main knowledge gaps to be addressed to improve smart maintenance of conveyor systems?

Regarding the eligibility criteria, only peer-reviewed articles published in English between 2010 and 2026 were included. Studies were required to focus on industrial belt conveyor systems, including the belt and related components such as idlers, rollers, pulleys, and drives. They also had to investigate anomaly detection, fault diagnosis, condition monitoring, or predictive maintenance using sensor data analyzed with machine learning, deep learning, computer vision, signal processing, or a combination of these methods. Review articles, theses, conference papers, and other non-peer-reviewed publications that were not peer-reviewed journal articles were excluded. We also excluded studies in which the monitored asset was not an industrial belt conveyor or one of its components (for example, generic bearing or motor studies, screw or scraper conveyors, or vibrating screens); conceptual studies without empirical evaluation; and studies relying only on manual inspection. Quantitative performance metrics (e.g., accuracy, precision, recall, F1-score, mAP: mean Average Precision at an IoU of 0.5, inference time) were not used to decide eligibility; they were extracted as study characteristics during full-text analysis and used only in synthesis. Methodological quality was not used to determine eligibility; all studies meeting the inclusion criteria were retained, and appraisal results were considered only when interpreting the findings. Reasons for exclusion at the full-text stage are summarized in the PRISMA flow diagram (Figure 1).

A comprehensive literature search was conducted across bibliographic sources, including IEEE Xplore, Scopus, the MDPI platform, and Google Scholar (searched with Publish or Perish) (Table S1). The final search was completed in September 2026. The search strategy combined three groups of terms with Boolean operators (AND/OR): (1) conveyor system: conveyor, conveyor belt; (2) problem type: fault, anomaly, damage, defect, tear, wear, crack, deviation, foreign object, overheating, stall, blockage, failure, rip, hazard; and (3) either a diagnostic or prognostic task (monitoring, diagnosis, detection, prediction, predictive maintenance, prognosis, remaining useful life, degradation, survival analysis) or a computational method (machine learning, deep learning, computer vision, segmentation, edge computing, IoT, digital twin, cloud, signal processing). Sensing technology was deliberately not used as a mandatory search term, and signal-processing and prognostic terms were included. This avoided excluding, at the search stage, studies that name a specific sensor or algorithm without using generic terms, as well as prognosis-oriented studies. Sensing modality was instead recorded during data extraction (Table 1). Our query retrieved 48 of the 80 studies included in [39] (60%), and 36 of these met our eligibility criteria and were included in the synthesis. Unlike our review, Rios-Colque et al. [39] also included conference papers, which fall outside our eligibility criteria; this difference in scope may partly explain the moderate retrieval rate.

A representative search query was formulated by combining terms from the three thematic groups: (“conveyor” OR “conveyor belt”) AND (“fault*” OR “anomal*” OR “damage*” OR “defect*” OR “tear*” OR “wear” OR “crack*” OR “deviation” OR “foreign object*” OR “overheat*” OR “stall*” OR “blockage*” OR “failure*” OR “rip” OR “hazard*”) AND ((“monitoring” OR “diagnos*” OR “detection” OR “prediction” OR “predictive maintenance” OR “prognos*” OR “remaining useful life” OR “degradation” OR “survival analysis”) OR (“machine learning” OR “deep learning” OR “computer vision” OR “segmentation” OR “edge” OR “IoT” OR “Internet of Things” OR “digital twin” OR “cloud” OR “signal processing”)). The query was adapted to the syntax and search fields available in each information source. Supplementary Table S1 summarizes the database-specific search fields, limits, yields, and implementation details.

All records identified through the search were imported into Zotero (V.10.0.5), where duplicate entries were removed. Study selection was then carried out in two stages. Titles and abstracts were first screened for relevance to industrial conveyor systems and intelligent maintenance. The remaining reports were sought in full text; reports that could not be obtained through institutional access were recorded as not retrieved. Retrieved reports were assessed in full text against the eligibility criteria (Table 1, Figure 1). Two authors (M.A. and R.D.) independently screened the records, and disagreements were resolved through discussion and consensus. Data from all included studies were extracted independently by the same two authors (M.A. and R.D.) using a standardized form. The two extraction sheets were then compared item by item, and disagreements were resolved by discussion before analysis. The extracted information covered study characteristics, the conveyor component or fault investigated, sensing modality, analytical method, deployment characteristics, reported performance, external validation, reproducibility, cost reporting, and advanced predictive capabilities (Table 1).

Variables that required judgment were coded, with explicit rules applied by both extractors. Real-time operation was coded YES when a study reported online operation or a processing time shorter than the acquisition interval; a camera frame rate alone was not sufficient. Cost reporting was coded YES for cost values or a quantified cost comparison, PARTIAL for qualitative discussion only, and NO otherwise. External validation followed appraisal domain D9 (Section 2.2), deployment was coded by where inference ran (local, edge, cloud, or hybrid), and advanced capabilities were recorded only when implemented, rather than discussed. Each study was assigned to one primary analytical category according to its main methodological contribution. These categories were deep learning (learned models such as CNN, YOLO, LSTM, GRU, or autoencoders, including those applied to images), machine learning (classical learned models such as SVM, random forest, decision trees, or k-nearest neighbors), computer vision (rule-based image processing without learned features), signal processing/NDT (hand-crafted signal analysis without a learned classifier), and physics-based monitoring (threshold rules or reliability models without a learned component). Because many studies combine approaches, all approaches used in each study were also recorded, giving the multilabel counts reported in Section 4.2. For the comparison of reported accuracy, the value retained for each study was the accuracy of the proposed model on its main test set.

This systematic review was not prospectively registered, and no formal review protocol was prepared before the study. The review questions (Q1–Q3), eligibility criteria, data-extraction form, and appraisal checklist with its scoring rules were defined before screening and were applied unchanged throughout the review. The completed PRISMA 2020 checklist is provided in the Supplementary Materials (Table S2).

### 2.2. Assessment of Methodological Quality

Quality assessment was performed using an adapted checklist based on Joanna Briggs Institute (JBI) criteria for appraisal of evidence [42]. To our knowledge, no JBI appraisal tool has been developed specifically for this type of engineering and AI validation study, so a tailored checklist was developed for this review. Because the checklist is adapted rather than externally validated, its results are reported as methodological concern rather than risk of bias. Ten domains were assessed: (D1) clear objective; (D2) appropriate study design; (D3) dataset description (source, size, class balance, acquisition conditions); (D4) ground truth (labeling procedure and fault confirmation); (D5) methodology described in sufficient detail; (D6) evaluation protocol (train/test separation, cross-validation, appropriate metrics); (D7) baseline comparison; (D8) statistical analysis (variance, confidence intervals, or significance tests); (D9) external validation (independent site, dataset, or operating condition); and (D10) reproducibility (data or code availability: YES when both code and data were publicly available, PARTLY when either was public or offered on request, and NO otherwise). Each domain was rated YES (2 points), PARTLY (1 point), or NO or UNCLEAR (0 points). The points were summed over the ten domains, giving a maximum of 20, and the total was expressed as a percentage of this maximum (Equation (1)).

(1) $$scre\left(\%\right)=100\times \left(2\times n\left(YES\right)+n\left(PARTLY\right)\right)/20;$$

Dataset description (D3), ground truth (D4), and evaluation protocol (D6) were defined as critical domains because weaknesses in them directly affect the reliability of reported performance. Overall methodological concern was then assigned as follows: (1) low, when the score was at least 80% and all three critical domains were rated YES; (2) moderate, when the score was between 60% and 79%, or at least 80% with a critical domain rated PARTLY or UNCLEAR; and (3) high, when the score was below 60% or any critical domain was rated NO. Two authors (M.A. and R.D.) assessed each study independently and resolved disagreements by consensus. Item-level ratings are provided in Table S3.

## 3. Quantitative Overview of Included Studies

### 3.1. Study Selection and Characteristics

Following the PRISMA 2020 protocol, the systematic search and screening process resulted in the identification of 131 empirical studies published between 2010 and 2026 that fulfilled the inclusion criteria for the review (Figure 1 and Figure 2). Publication activity has grown steadily: Notably, 123 studies (93.9%) appeared between 2019 and 2026, and nearly half of all included studies (*n* = 65, 49.6%) were published in 2024–2026 alone, compared with only eight studies between 2013 and 2018 and none between 2010 and 2012 (Figure 2A). This pattern suggests that research on intelligent conveyor belt monitoring has expanded mainly over the past few years and remains a relatively young field.

From the application perspective, mining accounted for most of the literature, with 97 studies (74.0%), followed by general industrial applications with 20 studies (15.3%). Manufacturing accounted for six studies (4.6%), ports and shipping for six (4.6%), and other application areas for two (1.5%) (Figure 2B). The predominance of mining reflects the importance of conveyor systems in continuous material handling operations and the operational consequences associated with belt failures.

### 3.2. Methodological Quality and Appraisal Concerns

Most studies were classified as having a moderate methodological concern, accounting for 76.3% (*n* = 100), followed by a high concern at 17.6% (*n* = 23) and low concern at 6.1% (*n* = 8) (Figure 3A). High concern ratings were associated with low normalized scores or failure in a critical domain, particularly ground-truth reliability or the evaluation protocol. These findings indicate that, although many studies reported clear objectives and methodological procedures, substantial weaknesses remain in validation, statistical robustness, and reproducibility.

All 131 included studies explicitly stated their research objective, and almost all presented an appropriate study design (*n* = 127, 96.9%) and methodology (*n* = 124, 94.7%) (Figure 3B). Comparison with baseline methods was reported in 104 studies (79.4%), a complete evaluation protocol in 98 (74.8%), and a full dataset description in 77 (58.8%) (Figure 3C). The main limitations concerned the quality of the evidence and reproducibility. Validation against a reliable ground truth was fully demonstrated in only 35 studies (26.7%), with partial evidence in a further 93; statistical analysis (for example, repeated runs, variance, or confidence intervals) was adequate in 16 studies (12.2%); external validation was fully met in 11 (8.4%), partly in 26, and not reported or unclear in 94; and reproducibility was fully met in only two studies (1.5%), partly met in 42 (code or data public, or data offered on request), and not met or unclear in 87. 

### 3.3. Validation and Deployment Characteristics

#### 3.3.1. Distribution of Validation Environments

Most studies evaluated the proposed methods under controlled conditions, with laboratory validation representing the largest category (*n* = 61, 46.6%; Figure 4A). Single-site validation was nearly as frequent (*n* = 59, 45.0%), indicating that a large part of the evidence comes from actual facilities, but generally in a single operational context. Examples among recent studies include full-line distributed optical-fiber monitoring of idlers in a coal-fired power plant [43], distributed acoustic sensing along a 2 km port conveyor [44], and a three-month IoT deployment in a coal mine [45]. In contrast, multi-site validation remains rare (*n* = 7, 5.3%); examples include six months of field data from crushers and conveyors [46] and underground video from several coal mines [47], and four studies (3.1%) combined laboratory and field data. The limited frequency of multi-site validation is a major constraint for generalization, as conveyor systems vary considerably from one facility to another in terms of belt type, operating speeds, loading regimes, lighting, dust levels, and visual expression or defect signals. Therefore, performance reported from a laboratory or single site may not be reliably transferable to unknown conditions without recalibration, domain adaptation, or additional site-specific data collection.

#### 3.3.2. Deployment Architecture

Reported deployment targets show a strong preference for on-premises processing, with local deployment accounting for 70.2% of studies (*n* = 92; Figure 4B). Edge deployment is the second most common configuration (*n* = 23, 17.6%), reflecting growing emphasis on low-latency inference and reduced bandwidth requirements in industrial environments; for example, distributed acoustic sensing data were compressed and classified close to the sensor [44]. Cloud-exclusive deployment (*n* = 3, 2.3%) and hybrid edge-cloud systems (*n* = 4, 3.1%) remain rare [29,48], while deployment is not reported in 6.9% of the studies (*n* = 9). This pattern suggests that the field is already oriented toward on-site execution (local or edge), but the documentation of architectural details, particularly end-to-end data flow and integration with maintenance systems, remains inconsistent.

#### 3.3.3. Implementation Cost Reporting

Economic feasibility is not sufficiently defined in the literature (Figure 4C). Only 29.0% of the studies include any form of cost discussion, of which 16.8% (*n* = 22) include comprehensive cost information and 12.2% (*n* = 16) include partial cost details. The majority of the studies (71.0%, *n* = 93) do not include any form of cost discussion. The decision to implement condition monitoring in an industrial setting relies on factors such as the cost of sensors, installation, hardware, and maintenance. When the cost or operational impact is discussed, the studies indicate that condition-based and predictive maintenance offer significant operational benefits, such as reduced unplanned downtime and improved scheduling efficiency, and thus advocate for the inclusion of economic reporting in future studies (e.g., [1,49]).

## 4. Sensing Modalities, Fault Types, and Modeling Approaches

### 4.1. Common Anomalies in Conveyor Systems

The 131 studies included in the review covered a wide range of conveyor anomalies (Table 2), with multi-fault detection being the most common category (69 of 131, or 52.7%) (Figure 3D). Mechanical failures were addressed in 60 studies (45.8%) and idler/roller faults in 50 (38.2%), making idlers and rollers the most frequently studied single component, followed by belt surface damage, including tears, cracks, scratches, and wear, in 45 studies (34.4%). Vibration abnormalities were reported in 29 studies (22.1%), acoustic anomalies in 22 (16.8%), belt deviation or misalignment in 23 (17.6%), thermal anomalies in 16 (12.2%), foreign objects in 15 (11.5%), and steel cord defects in 11 (8.4%) (Figure 3D). The literature indicates that sensor selection is typically based on the type of fault being monitored and the intended deployment conditions. This suggests that sensing strategies have evolved primarily through application-specific experience rather than from a common design framework.

#### 4.1.1. Belt Surface Damage and Cracks

Surface damage, mainly tears, cracks, scratches, wear marks, and breakage, is the main belt-related anomaly category [28,35,53,78]. Image-based inspection dominates here because of its information density and low instrumentation burden relative to the inspection area covered. Chen et al. [5] reported an mAP of 96.7% using a YOLOv8-based deep learning model adapted for real-time mining applications, and Wang et al. [73] developed a lightweight Single Shot MultiBox Detector (SSD)-based method to detect foreign objects, including bolts, waste steel, wood, and gangue, that can initiate longitudinal belt tearing, reporting 94.6% average detection accuracy at a processing speed exceeding 20 frames per second without GPU acceleration. Beyond visible surface defects, structural degradation at belt splices deserves more attention, since reduced splice strength can precede belt failure and is not always visible to surface-only inspection [12]. YOLO-based object detectors are common for surface damage detection, while segmentation networks are used when defect boundaries are needed for maintenance planning [79]. Detection accuracy is reported to depend heavily on defect diversity and environmental variability, and limited datasets remain a persistent constraint [34,36]. For small defects specifically, accuracy tends to fall, which is why several studies now favor multiscale architectures [80].

Recent studies have extended surface inspection beyond RGB images, mainly to obtain information that a two-dimensional image cannot provide. Depth sensing turns defect detection into a measurement task: the structured-light sensor of a smartphone and terrestrial laser scanning recover the geometry of cover defects and depressions [52,81], and a binocular line laser locates deviations and longitudinal tears from 3D point clouds in about 24 ms per image [82]. Belt flutter, which blurs laser-stripe images, was reduced mechanically with a spring damper before YOLOv7-ACmix crack detection [83]. Other studies addressed system integration and labeling cost: a YOLOv7 detector was embedded in a PLC-controlled laser scanning system [84], and a semi-supervised generative adversarial network was trained on 800 labeled and 2400 unlabeled images [85]. Several of these results were obtained on in-house datasets with induced or controlled defects, where scores close to the maximum are common. On a public benchmark and on four field hazard classes, mAP was lower, at 92.4% and 73.8%, respectively [54,86]. This gap suggests that part of the high performance reported for surface inspection reflects the narrowness of the test data rather than the method alone.

#### 4.1.2. Longitudinal Tear Detection

Longitudinal tear detection and early warning. Longitudinal tears, which can lead to catastrophic belt failure, have been studied using computer vision [15,78,80], thermal infrared imaging [50], and acoustic detection [51,87]. Laser-based methods are the most common choice for this fault type [18], though they require careful threshold calibration and may not generalize well without larger datasets or broader operating analysis [15]. Deep learning is increasingly used here too, improving reliability and enabling real-time localization [8], and an improved YOLOv7 applied to linear CCD images reached 94.6% precision at 110 FPS and was installed on a coal mine conveyor [88], but several studies still call for validation beyond controlled setups and under harsher environmental conditions [28,33].

#### 4.1.3. Idlers, Rollers, and Bearing Dynamics

Idler and roller faults, particularly bearing failures, form the largest component-specific anomaly category (*n* = 50, 38.2%). This fault family relies heavily on thermal, acoustic, and vibration sensing because early degradation manifests as a temperature anomaly or a temporal signature well before it becomes visible [2,13,20,21,69,89,90,91,92,93]. The danger extends beyond mechanical failure, since overheated idlers generate friction that can create fire hazards in continuous operation: Siami et al. [79] reported 99.9% accuracy for semantic segmentation of thermal defects in belt conveyor idlers, and Dąbek et al. [66] and Xiuyu et al. [67] highlighted the value of thermal monitoring for early fire risk detection in mining environments, where overheating in gearboxes, motors, and braking systems was effectively identified. Vision-based methods targeting rotation speed or visible end-surface cues are promising but often require visible or trackable features, which limits generalized deployment [22]. Recurrent models and hybrid architectures are repeatedly positioned as effective for time series data because they learn the temporal structure linked to incipient fault states [1,89], though datasets in this area are still collected mostly in laboratory simulations or single contexts, leaving open how well they hold up under field-level noise and operating variability.

For idlers, the trend is toward distributed and mobile sensing, which changes the question from whether a given roller is faulty to where along the line the fault is located. Distributed acoustic sensing and fiber Bragg grating arrays monitor many idlers along a single fiber. Liu et al. [43] applied this approach to a full conveyor line in a power plant and reported the variation across folds (90.3 ± 4.9%), whereas other studies reported single accuracy values between 96.7% and 100% [44,94,95,96]. Because the validation protocols differ, this spread says more about how the models were tested than about which signal-processing chain performs best. Two studies reduced the need for labeled faults, one through contrastive self-supervised learning with only 3.6% labeled data [70], and the other through semi-supervised acoustic anomaly detection [71]. Acoustic diagnosis was also addressed with a dual-stream attention network evaluated by five-fold cross-validation [97], and microphones are increasingly carried by inspection robots or drones instead of being fixed to the structure [98,99,100]. A masked-transformer autoencoder went beyond detection and localized the faulty idler on a test conveyor [72]. Thermal monitoring followed the same move from the laboratory to the site, with a customized ResNet-50 tested in an operating mine [68], and to low-cost IoT temperature nodes [101]. Mobile acquisition, however, introduces domain shift: when an acoustic model was transferred from handheld to drone recordings of the same installation, accuracy dropped to 86.0% [100], below the values usually reported within a single dataset.

#### 4.1.4. Belt Deviation, Misalignment, and Internal Steel Cord

Belt deviation and misalignment were addressed using computer vision techniques capable of detecting even slight changes in belt alignment before operational failures occur [55,102,103,104]. Zhang et al. [56] quantified conveyor belt deviation using image-derived lateral-offset and deflection angle measurements, with a laser-assisted computer vision implementation operating at approximately 26 frames per second in a laboratory conveyor setup. On the other hand, magnetic flux leakage and similar NDT techniques (e.g., X-ray) are still important when internal defects cannot be easily determined based on surface features [59]. Studies highlight practical constraints such as lift-off effects and sensor placement sensitivity and limitations tied to dataset size and class coverage [59,60]. Machine learning enhancement of NDT signals can improve classification, but the evidence reinforces that these tasks remain strongly constrained by sensing physics and experimental coverage [59,61,105].

Work on belt deviation has shifted from detecting misalignment to measuring it in physical units, which can be compared directly with alignment tolerances. DC-YOLO-seg estimated the offset in millimeters [106], an ABC-optimized adaptive segmentation quantified it with relative errors below 5% at 16.3 ms per frame [107], and attention-guided U-Net and fully convolutional models ran at 16 and 13.4 frames per second, respectively [57,108], so this fault family has evidence on both accuracy and processing speed. Validation has also started to extend beyond a single site: GES-YOLO was tested on a 5975-image dataset supplemented by videos from other mines [47], YOLOv5-based quantitative monitoring reached 67.7 frames per second [109], and GSSA-YOLOM handled deviation and foreign objects in a single network [74]. Learned camera models are not the only option. A classical vision pipeline and motor current signature analysis, which detects mistracking from the drive current without any camera, both reported high accuracy in their own test settings [58,110]. Sun et al. [111] forecast the deviation trend of a curved belt with an ARIMA–LSTM model, one of the few prediction-oriented studies in this category. Steel cord monitoring is moving in a similar direction. Repeated magnetic scans have been used to follow the growth of core damage over time [62], which is the kind of record that degradation models require, while other studies improved the signal chain by synchronizing multi-channel splice signals with a genetic algorithm [63] and denoising electromagnetic defect signals before classification [64].

Method dominance within any one fault family should not be read as universal superiority. The strongest systems treat sensing and modeling as a coupled design choice, selecting the simplest approach that is reliable for the specific fault physics and deployment context, rather than defaulting to whichever architecture is most reported.

### 4.2. Sensing Modalities and Analytical Approaches: Distribution and Trends

The distribution of detection approaches showed deep learning as the dominant technique (*n* = 72, 55.0%), followed by machine learning (*n* = 30, 22.9%), computer vision (*n* = 16, 12.2%), signal processing/NDT (*n* = 10, 7.6%), and physics-based monitoring (*n* = 3, 2.3%) (Figure 5A). These categories overlap in practice. Counting every approach used in a study, deep learning appeared in 83 studies, classical machine learning in 43, signal-processing or NDT feature extraction in 44, and vision data in 54; deep learning was applied to vision data in 41 studies and combined with a signal-processing front end in 23 (Table S4). Edge deployment describes where a system runs rather than an analytical method; it was reported in 23 studies and is analyzed with deployment (Figure 4B). This distribution traces a methodological trajectory from engineered, interpretable vision pipelines to feature-based machine learning and, more recently, to deep learning; each transition tracks a specific limitation of the stage before it rather than simply following a trend. Early studies relied on explicit computer vision workflows, preprocessing, edge/morphological operations, belt boundary tracking, and rule-based decisions that perform well when fault signatures are strongly geometric (e.g., deviation, rips/tears) and when labeled datasets are limited [6]. These methods remain attractive for safety-critical applications precisely because each processing stage is auditable and tunable, a property that end-to-end learned models trade away. Non-destructive testing and signal processing remain in use for the same reason, where interpretability, reliability, or sensing constraints dominate [43,58,64,112].

From a hardware perspective, vision/camera systems are the most widely deployed modality (*n* = 53, 40.5%), reflecting the flexibility of optical inspection for belt surface defects, alignment monitoring, foreign object detection, and operational state recognition (Figure 5B, Table S4). Multi-sensor fusion is also prominent (*n* = 27, 20.6%), indicating that a substantial share of studies integrates complementary signals (e.g., RGB + thermal, vibration + acoustic) to improve stability under industrial noise, dust, occlusion, and illumination variability (Table S4). Vibration (*n* = 16, 12.2%), acoustic (*n* = 13, 9.9%), and thermal/infrared sensing (*n* = 7, 5.3%) also show notable adoption (Figure 5B) but for different purposes: vibration sensing is used mainly for diagnosing rotating components such as idlers and bearings [21,65,69], while acoustic sensing provides non-contact measurement of internal mechanical irregularities [2,113]. Thermal/infrared imaging (*n* = 7, 5.3%) captures friction-related temperature increases and early bearing or idler degradation [17,68], and magnetic sensing (*n* = 7, 5.3%) is associated with steel cord integrity and internal defect assessment [59,62,105]. Electrical current or energy signals (*n* = 3, 2.3%) offer a sensor-free alternative based on existing drive data [58,77]. Distributed optical-fiber sensing (*n* = 2, 1.5% as primary modality, and used in several vibration-coded studies) is an emerging option for monitoring long conveyor lines with a single cable [43,44,70,94,95,96]. Pressure/force sensing (*n* = 2, 1.5%) was the least frequent sensing modality, and one study (0.8%) relied on maintenance records rather than sensor data.

### 4.3. Machine Learning Methods

Traditional machine learning (ML) approaches remain relevant, particularly when feature engineering is feasible, interpretability is prioritized, and when data are limited and informative feature sets can be extracted from vibrational or acoustic signals [3,20,61,114]. SVM-based models are the most frequently reported classical ML family [114], followed by decision trees and random forests (Figure 5D). Empirical performance reported in the extracted studies indicates that ML pipelines can achieve high accuracy in targeted settings (Figure 5D). Hao & Liang [115] reported 97.63% accuracy using a multiclass SVM for vision-based surface damage detection. Wang et al. [33] reported 98.40% accuracy for a machine vision tear detection pipeline built on classical learning components. In vibration-driven monitoring, Roos & Heyns [21] reported 100% accuracy in a belt vibration framework using wavelet packet decomposition-based feature representations. Li et al. [116] combined principal component analysis with a hybrid grey wolf optimized SVM to classify seven conveyor operating states from 19 IoT monitoring variables, reporting 97.22% test accuracy; however, this result was obtained from a test set of only 36 samples.

Feature selection also improved vibration-based idler diagnosis, for example, with decision trees and ANOVA [117] or a J48 classifier using four statistical features (89.5%) [118], and tree ensembles were used in IoT predictive maintenance systems [45,48,119]; LightGBM classified five conveyor states with 96% accuracy during a three-month deployment [45], and a grey-wolf-optimized multilayer perceptron on texture features classified large foreign objects with 98.8% accuracy and was field-tested in a coal mine [120]. Reports of 100% accuracy on small laboratory datasets illustrate the risk of optimistic estimates when test sets are small or not independent [48,119]. The main limitation of these methods is generalization: performance shifts with sensor configuration, belt speed/load, and environmental conditions [20], because the hand-designed features that make these models interpretable are also what tie them to the conditions under which they were engineered. Across the included studies, mean reported accuracy was similar for classical ML families (SVM, random forest, decision tree) and deep learning families (CNN, LSTM/GRU, autoencoder), between 92.9% and 97.4%, and the ranges overlapped widely (Figure 5D). The two families were, however, applied to different data. CNNs were mainly applied to images and classical models mostly to multi-sensor, acoustic, and vibration signals, and none of the included studies compared them head-to-head on the same task. These findings show that classical ML can remain competitive for well-defined fault classes with informative engineered features, without establishing cross-study superiority over deep learning (Figure 5A). For Q2, the evidence therefore supports choosing between them by signal type and available training data rather than ranking one above the other.

### 4.4. Deep Learning Approaches and Architecture

Deep learning (DL) was the most common analytical method in our evidence base (Figure 5A; 55.0% of studies) because it can handle noisy and varying operating conditions, such as illumination changes, background clutter, and subtle or low-contrast defects, by learning features directly from raw industrial data rather than relying on hand-designed descriptors [5,8,121]. It has become dominant mainly because it reduces dependence on manual feature engineering and tolerates image variability that would otherwise require re-engineering features for every new site. End-to-end representation learning matters especially in conveyor systems, where signals are noisy and defect presentation shifts with operating conditions, including early mechanical degradation that first manifests as subtle temporal patterns in acoustic and vibration signals [13,89,113]. DL offers a framework that can handle diverse modalities (RGB/thermal imaging, vibration, acoustic, and multi-sensor data) and supports real-time decision-making pipelines for safety-critical monitoring and downtime prevention (Figure 6A) [33,79,122].

Model selection in the reviewed studies is strongly dictated by data structure. For vision-based inspection, convolutional architectures dominate. YOLO family detectors, particularly, are now used routinely for real-time anomaly detection on conveyor images because they balance accuracy and latency, enabling real-time localization of surface damage, tear cues, and foreign objects [75,123,124,125,126], including under fog and motion blur in coal mine video [127,128]. For example, Zhang et al. [126] combined EfficientNet with YOLOv3 to detect four types of mining conveyor belt damage, achieving an mAP of 97.26%. ResNet/VGG backbones are commonly applied to defect classification, and encoder–decoder designs such as U-Net support segmentation when pixel-level localization is required to guide maintenance actions, such as mapping defect extent or hotspots. These choices are consistent with the prominence of camera-based monitoring in the literature (Figure 5B), where imaging offers dense, cost-effective coverage of belt surfaces and operating states. The same studies that report this performance, however, also report the conditions under which it was obtained: Chen et al. [5] note a limited range of belt types and damage diversity in their evaluation; Wu et al. [28] flag the need to test performance under harsher operating conditions. Also, Yuan et al. [36] and Zhao et al. [34] identify computational complexity as a constraint on integrated deployment. The accuracy gains attributed to deep learning are therefore real within the tested conditions, but the tested conditions are narrower than the operating envelope of an actual mine or port, a gap this review returns to in Section 5.

Recent studies have also begun to report results on public benchmarks or across sites [47,54,74]. On these data, mAP values (73.8–92.4%) [54,74,86] are lower than those usually reported on in-house datasets, which supports the view that benchmark diversity, rather than architecture alone, determines how far reported accuracy can be trusted. Hybrid architectures that couple convolutional and recurrent or attention layers are now common for acoustic and vibration data [46,96,97], and transformer-based models have appeared for both vision [129] and multi-channel acoustic–vibration monitoring [72].

For monitoring dominated by time-series data, especially idler/bearing and rotating component diagnostics, recurrent networks (LSTM/GRU) and hybrid CNN-RNN pipelines are frequent because diagnostic information is often embedded in temporal evolution rather than static appearance (Figure 5D, Figure 7). These architectures suit early warning, where subtle temporal deviations can precede visible damage [37,89,124]. When labeled fault examples are inadequate, autoencoder-based approaches provide a practical alternative. By learning “normal” behavior and flagging deviations via reconstruction error, these models enable data-efficient anomaly scoring and can generalize to unknown or emerging failure modes [26,72,130]. Semi-supervised and self-supervised learning pursue the same goal of reducing labeling effort, for example, with chroma-augmented one-class anomaly detection [71], generative adversarial training on partly unlabeled images [85], and contrastive pretraining that needed only 3.6% labeled data [70]. DL also extends beyond binary detection toward actionable localization, for example, through thermal semantic segmentation of overheated idlers [79], audio–visual localization of faulty idlers (mIoU = 73.8%) [131], and emerging dataset contributions aim to improve benchmarking comparability across studies [34,36].

### 4.5. Computer Vision-Based Monitoring

Computer vision (CV) remains a major methodological stream in conveyor condition monitoring (Figure 5A; 12.2% of studies), offering interpretable, engineering-driven pipelines that extract diagnostic cues from images through photometric and geometric processing rather than fully end-to-end learned representations. Across our evidence base, CV is most frequently associated with vision/camera acquisition and is increasingly extended to thermal infrared and laser/structured light systems to capture failure signatures that may be weak in RGB alone or that require higher geometric precision [14,17,78].

A typical computer vision workflow maps to four operational stages (Figure 7). (1) Image acquisition uses fixed RGB cameras, thermal infrared systems, or laser-based scanning for surface geometry [6,78]. (2) Image enhancement applies denoising, illumination compensation, and contrast normalization to improve stability under dust, fog, and variable lighting [17,33]. (3) Feature extraction and geometric reasoning identify belt edges, centerlines, and defect regions through edge detection, morphology, segmentation, region of interest definition, and tracking [15,103]. (4) Decision rules or trained classifiers convert these features into alarms using shape, threshold, and temporal consistency criteria.

It is also noteworthy that the technique of CV is particularly effective for faults that have considerable geometric structure. Initial online machine vision techniques showed the feasibility of belt inspection in actual operating environments [6], followed by improved longitudinal tear detection using algorithmic and geometrical techniques such as structured illumination and laser techniques [15,78]. Most importantly, the technique of CV is also effective in situations of low visibility. Detection of tears in harsh environments (uneven illumination and complex backgrounds) is an important example of the effectiveness of the technique [33]. Similarly, belt deviation detection is naturally aligned with boundary tracking and geometrical constraints, achieving near-ceiling performance in controlled environments [103]; a classical pipeline based on improved Canny edge extraction and Hough line fitting reached 95.4% accuracy in field tests [110]. Three-dimensional acquisition extends this geometric reasoning to cover depressions and edge irregularities [52].

Thermal CV extends monitoring to fault modes that are not reliably detectable in RGB, such as overheat faults caused by friction or idler/bearing degradation [66]. It also aids in developing scalable acquisition concepts such as UAV-based thermography or robotic/UGV acquisition, as discussed in Carvalho et al. [14] and Siami et al. [17,132]. A legged inspection robot has also recorded belt edges to detect damage and deviation in an underground mine [133]. Lastly, multimodal computer vision, which combines RGB and thermal images and sometimes laser geometry, improves robustness by drawing on additional information when it is available [22,79].

### 4.6. Edge Computing and IoT Architecture

Edge computing and IoT architectures translate algorithmic advances into deployable conveyor monitoring by addressing three persistent field constraints—latency, bandwidth, and intermittent connectivity—that limit cloud-only solutions; in many safety-critical contexts, sub-second response is required to reduce downtime and enable timely intervention, consistent with the high proportion of studies reporting real-time operation (56.5%, Figure 5C). In our evidence base, edge deployment was reported in 23 studies (17.6%) and hybrid edge–cloud deployment in four (3.1%) (Figure 4B), indicating that a distinct subset of the literature prioritizes architecture and deployment (edge preprocessing/inference near the belt, event-driven transmission, and cloud storage/analytics) rather than offline benchmarking alone [24,25,48,80,101,134].

The most common architecture is based on a layered pipeline (Figure 7). The physical layer comprises the instrumented components of the conveyor system, such as the belt, rollers, and drive systems, which are equipped with one or more detection modes, most often cameras, but sometimes accompanied by vibration/acoustic or temperature sensors when early warning is desired [76,135]. The edge layer is responsible for controlling acquisition, preprocessing, and local inference, converting high-throughput raw data into a dense set of decision artifacts such as defect class, confidence level, timestamp, and the image of the area of interest, which can be transmitted in real time over the network. The cloud/server layer is used for historical recording, dashboards, reporting, and sometimes retraining and version management, as shown in recent studies [27,29,45].

The reviewed studies highlight diverse but convergent hardware patterns for edge inference. GPU-enabled embedded devices are frequently used when high-resolution vision workloads must run online; for example, Jetson-class deployment is reported for real-time object detection pipelines optimized for embedded execution [80], including foreign-object detection on a Jetson Xavier NX [120,128]. Other systems prioritize reliability and deterministic integration by coupling industrial PCs with PLCs (Programmable Logic Controllers), enabling continuous monitoring with local control actions and robust industrial interfacing [134]. There are also sensor-centric edge nodes, which include Arduino/MCU-based systems for distributed monitoring, where cost, power, and ease of installation are the dominant constraints for the design [24,104]. Generic commercial hardware, such as industrial PCs or high-power GPUs, is often too costly or poorly suited to harsh operating conditions (dust, vibration, temperature extremes), and developing custom acquisition nodes that combine efficient local computation with low-power, long-range connectivity (e.g., NB-IoT/LTE-M-class cellular modules) is a promising direction for addressing the transfer and multi-site scalability challenges raised throughout this review. In the context of monitoring rotating components, sensor nodes embedded close to the bearings/idlers demonstrate the use of IoT technology in facilitating in situ condition monitoring [135].

Model design is closely related to edge feasibility. Several studies have focused on hardware-aware efficiency, using efficient design and runtimes to meet hardware constraints without compromising operational usefulness. This includes “hardware-friendly” detection pipelines for operational on-belt inference [56], as well as edge-aware implementations on industrial PC/GPU platforms [53]. Communication is typically event-driven rather than using full-resolution streaming, reducing the load and maintaining traceability and operational maintenance signals [27,134]. Compressing raw sensor streams before transmission follows the same logic, for example, by converting distributed acoustic sensing data into compact cepstral features for edge classification [44].

The analysis in this section points to a clear answer to Q2: no single technique offers the best trade-off for every case. The best balance between accuracy, speed, and deployment feasibility comes from matching the model to the signal and the fault physics. For camera-based monitoring of surface damage, tears, belt deviation, and foreign objects, lightweight single-stage CNN detectors are the most promising option [5,8,47,126]. They combine high detection performance with real-time inference and are the only learned models already shown to run on embedded GPUs close to the belt [80]. Rule-based vision pipelines remain a sound choice when the fault signature is geometric and labeled data are scarce, because they run on modest hardware and every processing step can be audited [103,110]. For idlers, bearings, and drives monitored through vibration, acoustic, or electrical signals, feature-based machine learning and signal processing provide the most hardware-efficient solutions [2,21,58], and suit low-power edge nodes [24,45]. Recurrent, attention-based, and autoencoder models offer advantages in early warning and in learning from limited fault labels [46,70,71,96], but at a higher computational cost. On deployment, the evidence favors on-site processing: local or edge inference satisfies the latency and connectivity constraints of conveyor operations [44,80,134], and the cloud is better suited to storage, fleet-level analytics, and model updating than to real-time detection [27,29,48]. These conclusions remain conditional, because the included studies rarely report accuracy, latency, hardware, and energy use for the same deployed system, and most were validated in a single setting [34,36,100]. The most promising techniques are therefore those already proven for a given signal type; whether their performance holds across sites remains largely untested.

## 5. Critical Analysis and Research Gaps

### 5.1. Evidentiary and Reporting Gaps

Individual studies report strong performance in controlled settings: deep learning achieves high accuracy in belt damage detection [5,28], thermal segmentation [79], and reconstruction-based anomaly scoring [26,72]. Set against the appraisal in Section 3.2, this needs a caveat: only 6.1% of studies were rated as having low methodological concern, ground-truth reliability was fully demonstrated in 26.7%, and statistical analysis was adequate in just 12.2%. Two similar accuracy figures are not necessarily comparable if one study reports how its ground truth was established and the other does not.

Validation shows this most clearly: laboratory (46.6%) and single-site (45.0%) settings dominate; multi-site validation appears in only 5.3% of studies; and facilities vary widely in belt material, speed, load, dust, and lighting [34,36]. Reported accuracy may therefore not transfer without recalibration, which argues for multi-site, longitudinal validation across belt aging and seasonal conditions. Deployment reporting follows the same pattern: Notably, 56.5% of studies claim real-time operation, but communication protocols, PLC/SCADA integration, and alerting logic are often omitted, so systems with similar accuracy may differ sharply in operational value without this being visible in the literature. Explicit reporting of compute placement, runtime constraints, and alert logic would close this gap. Cost and reproducibility fare worse: Overall, 71.0% of studies give no cost information, source code is available for only 3.8%, and public datasets for 6.9% (Figure 5C), leaving procurement comparisons and independent verification largely infeasible.

### 5.2. Scientific and Technical Research Gaps

The gaps above concern how existing studies were designed, validated, and reported. A different question is whether the underlying modeling techniques have kept pace with what industrial deployment actually needs. We examined the full extraction dataset for seven capabilities that adjacent prognostics and health management research already treats as standard: remaining useful life (RUL) estimation, physics-informed modeling, principled sensor fusion architectures, domain adaptation for cross-site transfer, calibrated uncertainty quantification, explainable AI, and digital-twin integration (Table 3, Figure 6B). Two are entirely absent from the 131 studies: physics-informed machine learning and explicit cross-site domain adaptation. The others are present but rare: RUL estimation in three studies (2.3%), degradation trajectory or trend forecasting in seven (5.3%), principled multi-sensor fusion in three (2.3%), predictive uncertainty quantification in one (0.8%) without demonstrated calibration, post hoc explainability in six (4.6%), and digital-twin integration in three (2.3%). Broader, related practices are more common but do not meet these definitions: 18 studies (13.7%) include a physics-based or NDT component, 27 (20.6%) use several sensors, and 18 (13.7%) use generic transfer learning or pretraining.

The search, which explicitly included prognostic terms, therefore found a small number of prognosis-oriented studies, but it is methodologically diverse and largely outside mainstream deep learning. It includes probabilistic hidden Markov models for diagnosis and RUL estimation of low-speed idler bearings [136], bearing-life and failure-rate calculations driven by measured field loads [137], damage-growth forecasting from repeated magnetic scans to schedule belt refurbishment [49,62], trend prediction of belt deviation [111], neural-network forecasting of belt repairs and cable cuts from mine maintenance records [138], and a digital-twin framework that combines machine learning classification with RUL estimation for logistics equipment [29]. Elahi et al. [77] showed that gradual loss of belt tension can be identified early from motor energy data but framed the task as classification rather than remaining-life estimation. In most of these studies, the prognostic output is a calculated or forecast service life rather than a prediction validated against observed failures, so the accuracy of these estimates remains largely unknown. Similarly, the closest approach to cross-site transfer adapted an acoustic model from handheld to drone recordings of the same installation [100], rather than between different sites, and one digital-twin study relied on simulated fault data [30].

Degradation state-space models offer a more structured route from these indicators to prediction. In machining, Umar et al. [139] modeled tool-wear evolution as a degradation state constrained to remain consistent with observed failure and used it to estimate remaining useful life. In such models, an unobserved degradation state evolves over time according to a degradation law, measured features are linked to this state through an observation model, and failure is defined as the state crossing a threshold consistent with observed failures. The state and its uncertainty are updated recursively as new measurements arrive, for example, with Kalman or particle filtering, so that the RUL estimate is accompanied by a confidence interval. Several conveyor indicators already reported in the included studies fit this structure: cover-thickness loss from profile or laser scans, steel-cord damage density from successive magnetic inspections [62], and vibration or acoustic health indicators of idler bearings [136,137]. Applying these models would require run-to-failure or replacement records to set failure thresholds, which are rarely reported in the conveyor literature; building such datasets is therefore a precondition for moving from detection to calibrated prediction.

Independent support for this gap analysis comes from several review works (e.g., [38,39,40]), despite their different scope and methods. Rios-Colque et al. [39], the closest in scope to the present review, similarly identify the need to move beyond reactive fault detection toward systems that estimate remaining useful life. The present analysis extends that discussion by quantifying whether specific technical capabilities required for this transition are already represented in the evidence base. Rzeszowska et al. [38] devote a dedicated section to explainable AI and digital-twin integration as priority directions. Rojas et al. [40] raise explainability as an open research question and mention physics-informed prognostics in passing. All three also discuss multi-sensor fusion, but as a growing and beneficial trend rather than an architecture that needs to become more principled, which is the more specific claim made in Table 3. None of the three identify the absence of calibrated uncertainty quantification. On these last two points, the gap analysis here extends beyond what has previously been reported in the literature.

The main modeling priorities are summarized in Table 3 and require new scientific development rather than improved reporting alone. Five priorities emerge: first, linking detection to prognosis through degradation-trajectory and RUL-estimation models built on existing field outputs and validating these estimates against observed failures, which the few existing prognostic studies rarely do [29,136,137]; second, incorporating physics-informed constraints and mechanistic models of belt fatigue, splice degradation, and steel cord wear so that detectors can extrapolate beyond the conditions on which they were trained; third, replacing feature concatenation with fusion architectures that explicitly weight the reliability of each modality, a shift that presupposes hardware-level temporal synchronization across modalities: without precise source timestamping, alignment between acoustic, thermal, and visual streams remains approximate, which limits how much any fusion architecture can ultimately achieve; fourth, domain adaptation methods to reduce performance losses when models are transferred between sites, rather than relying on pre-trained weight initialization alone; and finally, integrating uncertainty quantification and explainability into detection pipelines, and where appropriate into digital-twin or system-level models, so that outputs can be trusted, interpreted, and acted upon rather than merely reported.

Work on other industrial assets suggests ways to address some of these gaps. For milling-machine fault diagnosis, Ullah et al. [31] combined CWT-based image augmentation, a modified AlexNet, ant-colony feature selection, and SVM classification, which is one way to obtain informative feature representations. Haneef et al. [32] address the uncertainty gap more directly: they applied residual conformal monitoring to wind-turbine SCADA data, with a focus on alarm calibration and on evaluation across assets under distribution shift. Neither approach has yet been tested on conveyor systems, but both are worth investigating as ways to improve diagnostic reliability, uncertainty calibration, and cross-site generalization.

This review has five main limitations. First, the search was limited to English-language, peer-reviewed journal articles found in IEEE Xplore, Scopus, and the MDPI platform, supplemented by Google Scholar. Relevant work published in other languages, notably Chinese-language mining journals, or outside these sources may have been missed. Restricting the review to peer-reviewed journal articles excluded conference proceedings, potentially overlooking relevant methodological advances, including those reported in conference papers cited in the closely related systematic review. Second, 39 of the 214 reports sought could not be retrieved in full text. Third, the review was not registered, and the search strategy was refined and rerun after an initial analysis to improve its sensitivity to signal-processing and prognosis-oriented studies. Although the eligibility criteria, extraction form, and appraisal rules were kept unchanged and the refined query only broadened the search, this sequence may have introduced some post hoc decision bias. Fourth, the outcomes and metrics reported in the studies were too heterogeneous to combine in a single statistical analysis, so the synthesis remained descriptive, and no meta-analysis was conducted. Finally, methodological quality was assessed with a checklist developed for this review rather than an externally validated tool, and the coding of advanced capabilities (Table 3) relies on what the studies reported. Although ratings were assigned independently and resolved by consensus, some subjectivity remains in the appraisal.

## 6. Conclusions

This review shows that intelligent fault detection for belt conveyor systems has advanced substantially between 2010 and 2026, with strong technical potential across a broad range of sensing and modeling approaches. Across 131 empirical studies, detection performance is often strong, and deep learning now dominates the field: CNN-based models were the most frequently reported approach for vision-driven tasks such as surface damage, tear hazards, belt deviation, and foreign object detection, while temporal architectures such as LSTM and GRU were mainly applied to acoustic and vibration time series. Distributed optical-fiber sensing has emerged as a practical option for monitoring idlers along entire conveyor lines. No single sensor or algorithm is universally optimal; reliable performance instead depends on matching sensing modality to fault physics: vision or laser systems for surface defects; thermal sensing for overheating and fire risk; acoustic, vibration, or distributed fiber sensors for rotating components; and magnetic or other NDT methods for internal steel cord and splice inspection. Beyond this technical synthesis, the review’s more significant finding is that the field has optimized detection while leaving prediction largely unexplored: only three of the 131 studies estimate remaining useful life and seven forecast degradation trends; physics-informed machine learning and cross-site domain adaptation are absent; and principled sensor fusion, calibrated uncertainty, explainability, and digital-twin integration each appear in at most six studies; predictive uncertainty was quantified in only one study, without demonstrated calibration.

The field should not yet be considered mature from an industrial deployment perspective, and the path forward has two distinct components. Most studies (91.6%) still rely on laboratory or single-site validation, and decision-critical reporting on false alarms, latency, computational burden, uncertainty, reproducibility, and cost remains inconsistent. This gap can be closed largely by the adoption of validation and reporting practices that are already supported by the field. The more significant issue concerns the underlying science: moving from detection to prediction will require modeling of degradation and remaining useful life, physics-informed constraints, principled multi-sensor fusion, and calibrated explainable inference.

## Figures and Tables

**Figure 1:**
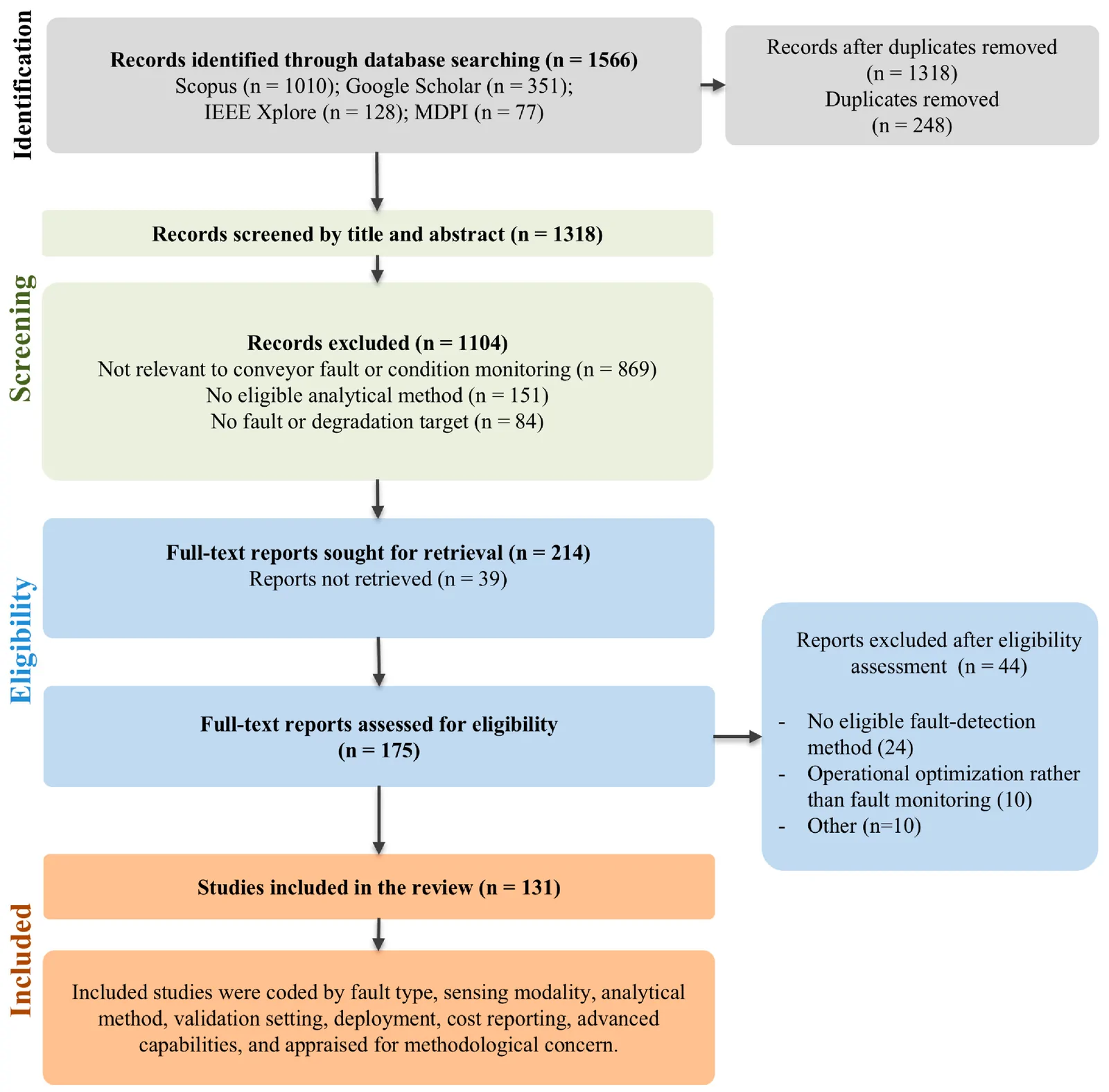
PRISMA 2020 flow diagram of the study selection process. Of 214 reports sought for retrieval, 39 were not retrieved; 175 underwent full-text assessment, 44 were excluded, and 131 studies were included. The Other category (*n* = 10) includes studies with unrelated monitoring targets, non-belt conveyor systems, or an ineligible publication type.

**Figure 2:**
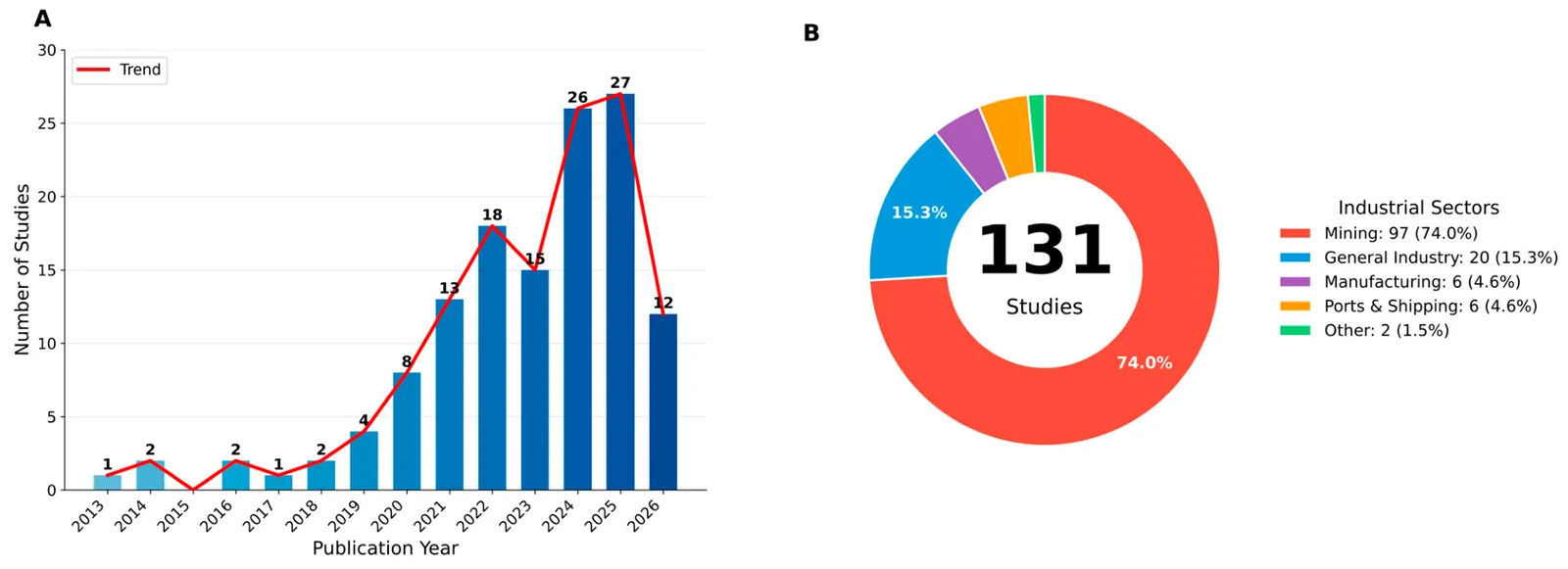
Study distribution by time and sector (*n* = 131). (**A**) Publications per year with fitted trend line. (**B**) Industrial application sector distribution (% of studies).

**Figure 3:**
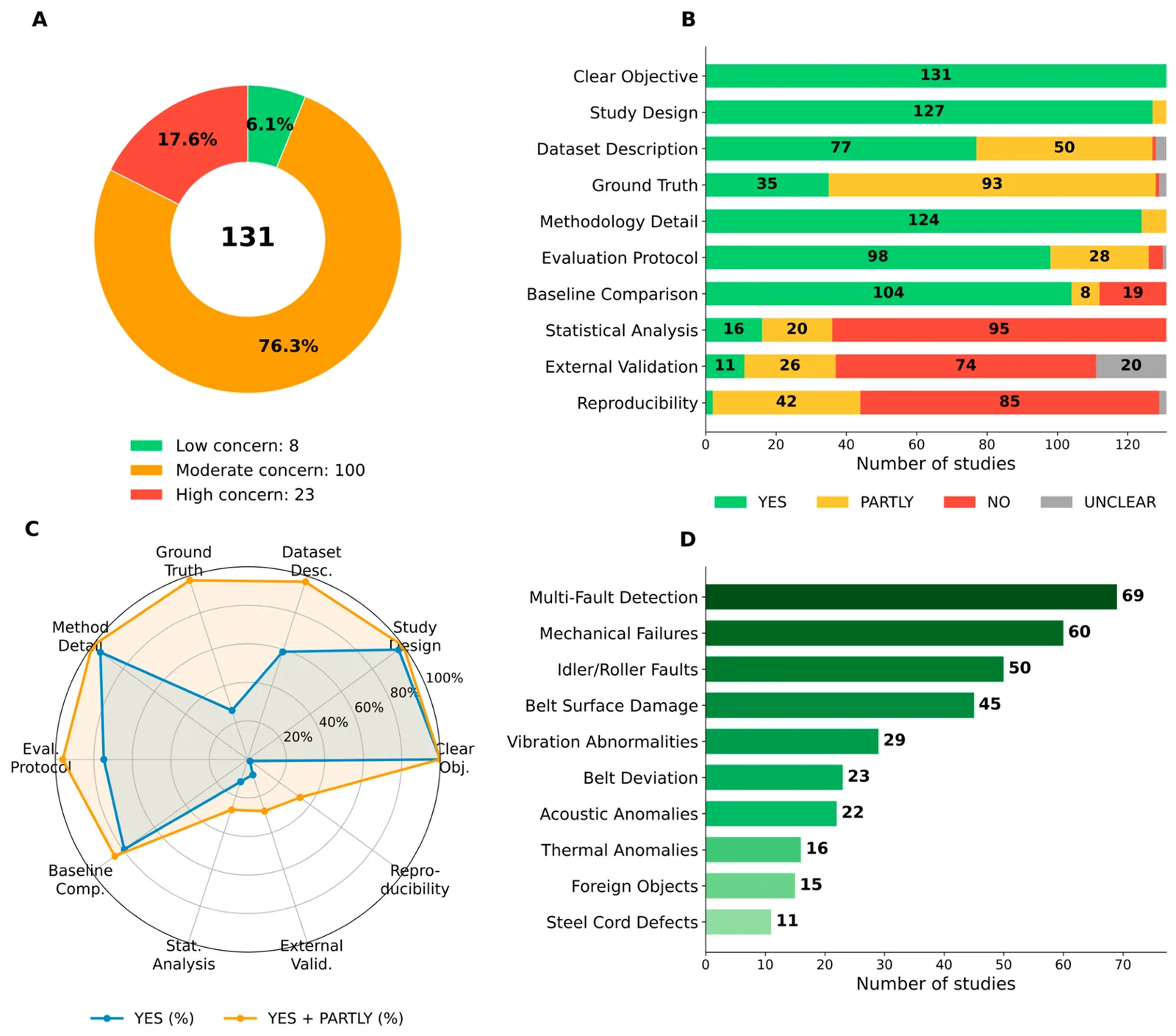
Methodological quality and anomaly coverage of included studies (*n* = 131). (**A**) Overall methodological concern: low, 6.1% (*n* = 8); moderate, 76.3% (*n* = 100); and high, 17.6% (*n* = 23). (**B**) Domain-level ratings (YES, PARTLY, NO, UNCLEAR) for the ten appraisal domains. (**C**) Radar chart of the percentage of studies rated YES and YES or PARTLY in each domain. (**D**) Frequency of anomaly types addressed in the reviewed literature; studies could report multiple anomaly categories. Study-level methodological appraisal ratings are provided in Table S3.

**Figure 4:**
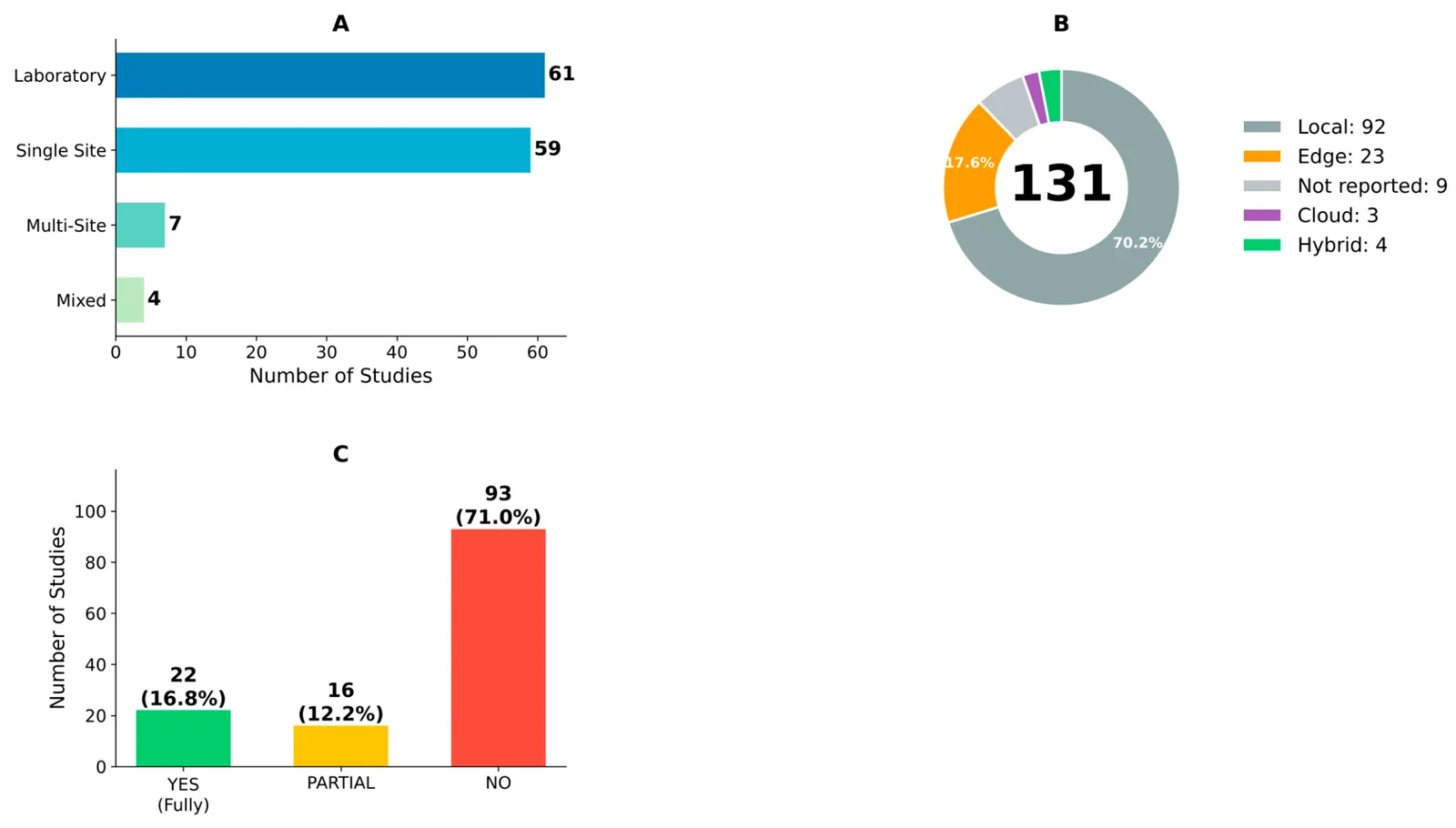
Validation setting, deployment architecture, and cost reporting across included studies (*n* = 131). (**A**) Validation environment used in empirical evaluation (laboratory, single site, multiple sites, or mixed laboratory + site). (**B**) Deployment type of the monitoring/analytics system (local/edge/cloud/hybrid, or not reported). (**C**) Reporting of implementation cost information, categorized as fully reported (YES), partially reported (PARTIAL), or not reported (NO).

**Figure 5:**
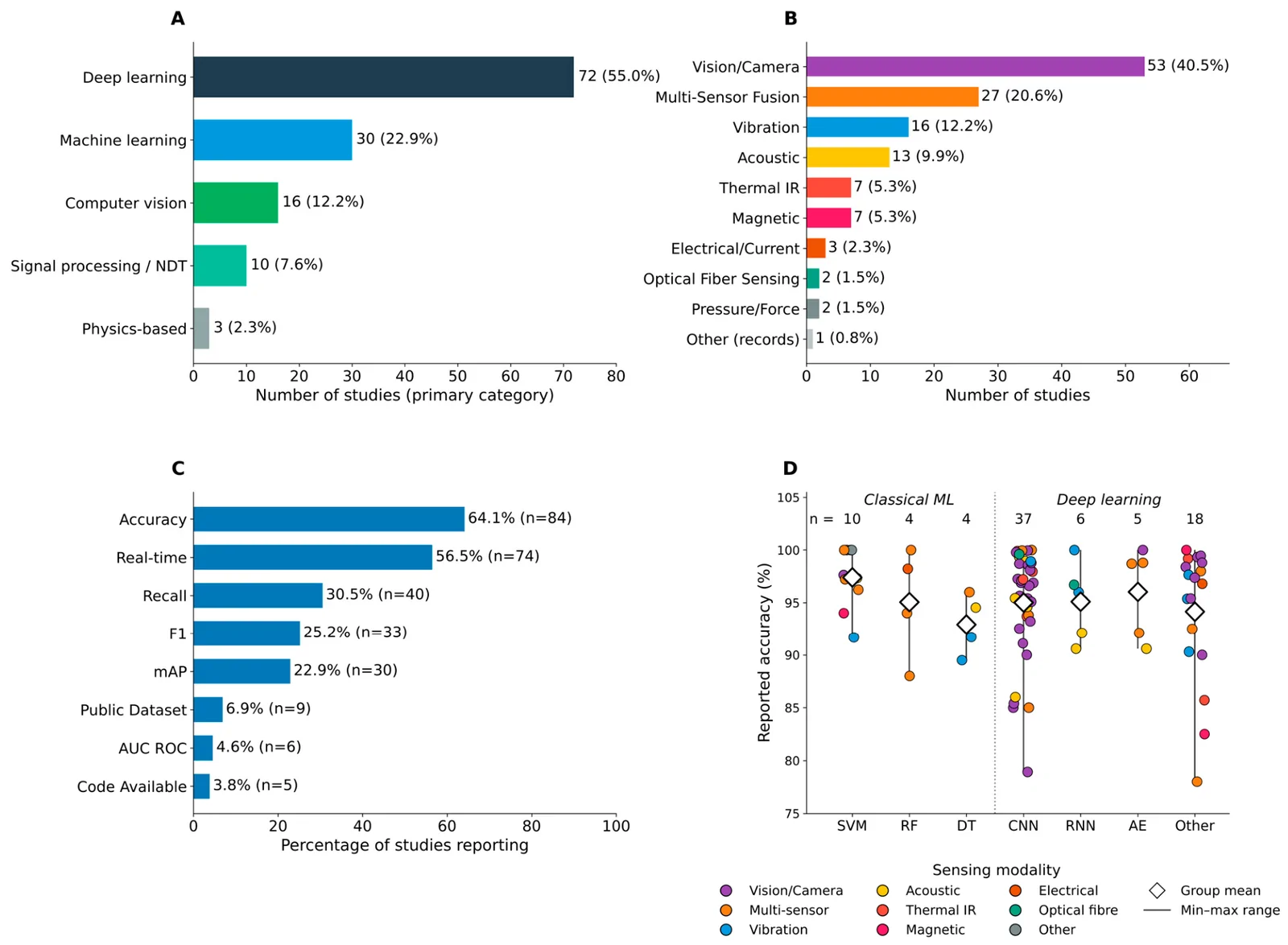
Methods, sensing modalities, and performance reporting in the included studies (*n* = 131). (**A**) Distribution of primary detection/analysis approaches. (**B**) Distribution of sensor modalities used for conveyor monitoring. (**C**) Reporting frequency of performance and implementation indicators (percentage of studies reporting each item). (**D**) Reported accuracy by main algorithm (one point per study, *n* = 84; RNN = LSTM/GRU, AE = autoencoder; color indicates sensing modality; diamonds give the group means and lines the minimum–maximum range). Values come from different datasets, tasks, and validation settings and are descriptive only.

**Figure 6:**
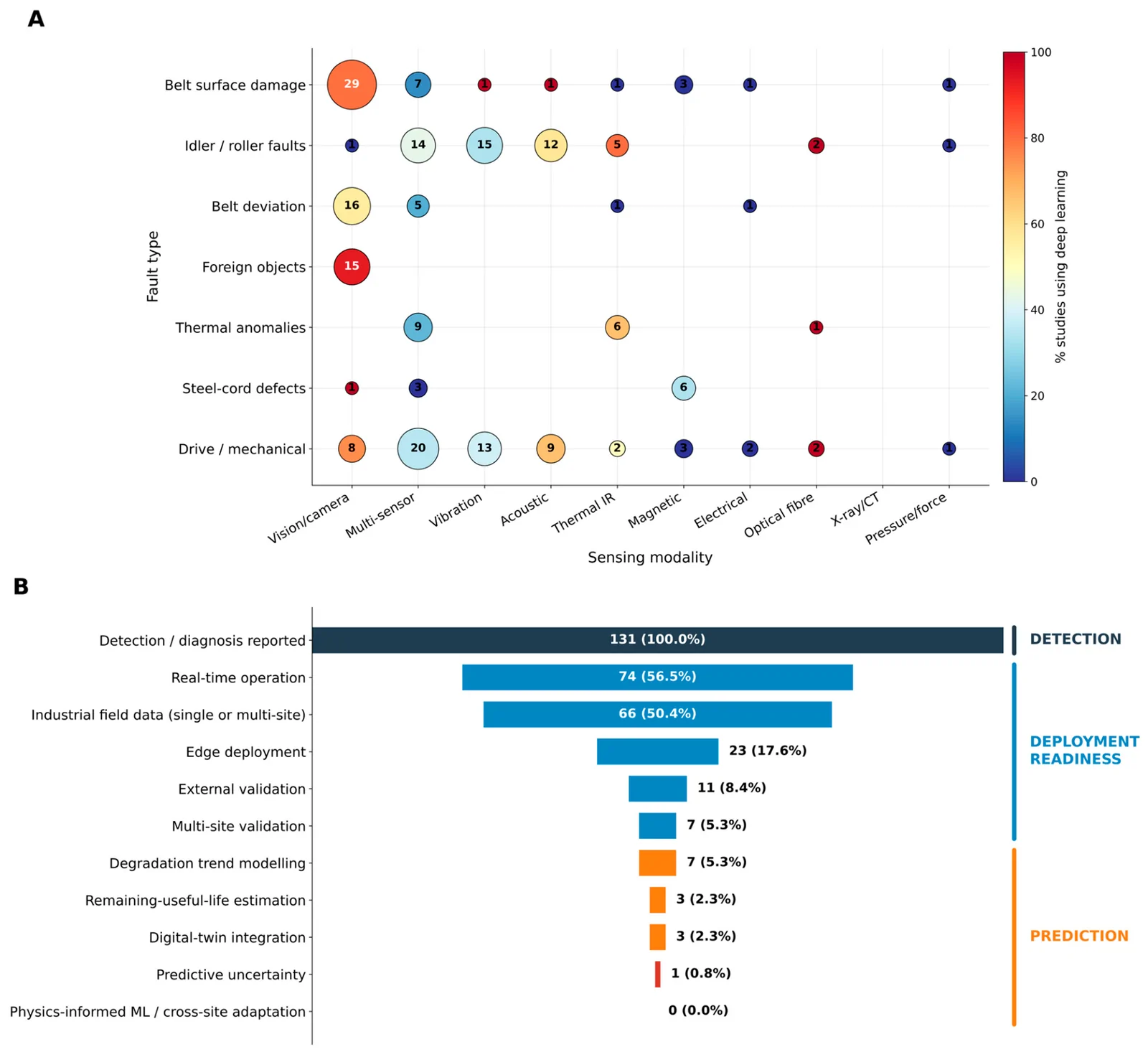
(**A**) Distribution of the included studies by fault type and sensing modality; the color scale indicates the proportion of deep-learning approaches. (**B**) Number of studies (*n* = 131) reporting each capability level, from detection to prediction.

**Figure 7:**
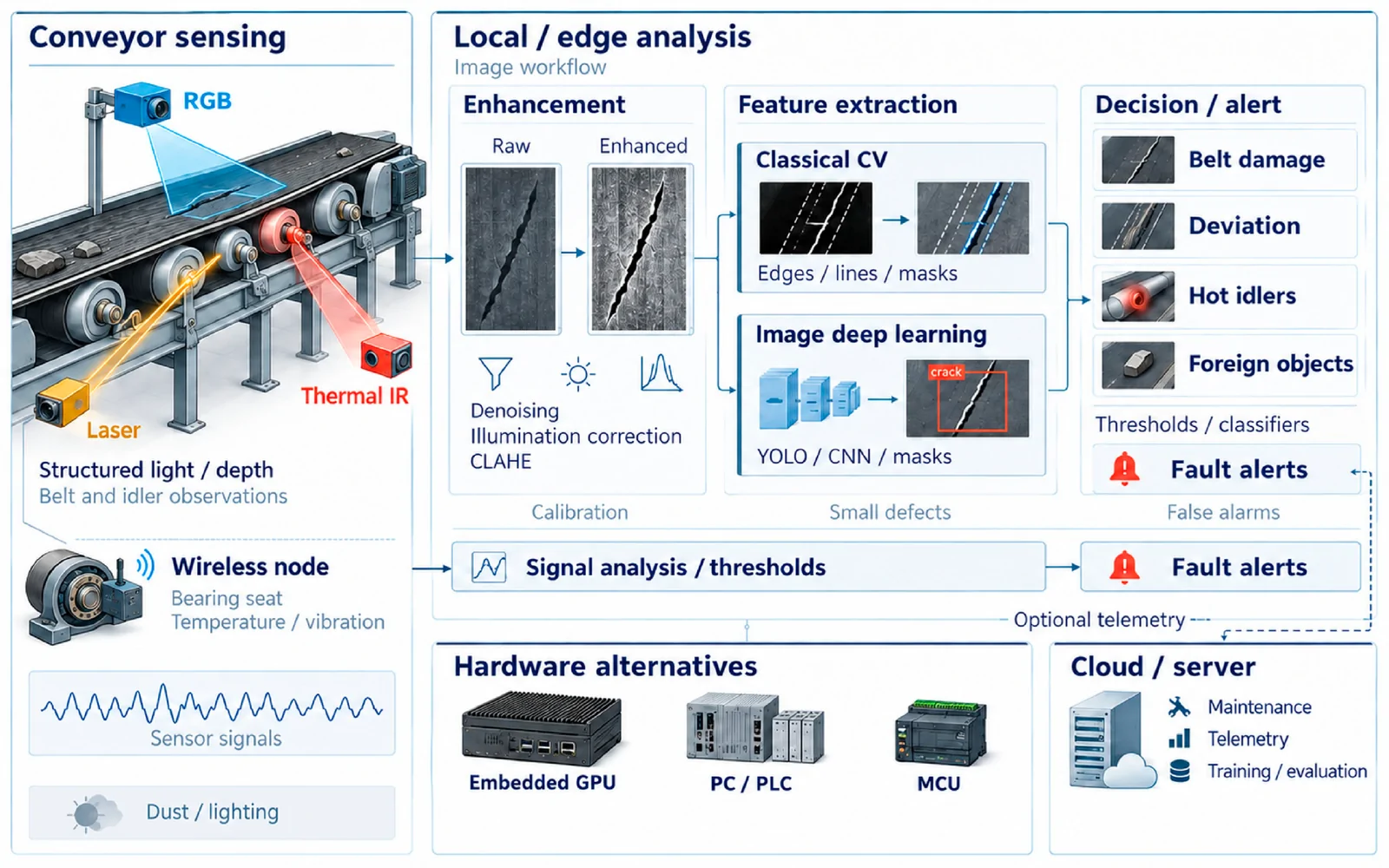
Integrated conveyor condition-monitoring workflow. Schematic synthesis of sensing, image and signal analysis, local/edge processing, fault alerts, and optional cloud/server functions reported across independent studies (e.g., [14,25,27,46,98]; designed by the co-authors). GPU: graphics processing unit; PC: personal computer; PLC: programmable logic controller; MCU: microcontroller unit; SCADA: supervisory control and data acquisition; CLAHE: Contrast Limited Adaptive Histogram Equalization.

**Table 1:** Summary of the structured data extraction categories applied to all 131 included studies following PRISMA 2020 guidelines.

Category	Extracted Data
**Study Descriptors**	Year, venue, industrial sector, laboratory vs. field context
**Targets (Q1)**	Conveyor component(s) and anomaly type(s) (e.g., idlers/rollers, belt surface damage, steel cord defects, mistracking, foreign objects, thermal anomalies)
**Sensing (Q1)**	Modality (vision/camera, thermal/IR, acoustic/ultrasound, vibration, magnetic/inductive, pressure/force, electrical current, distributed optical fiber, X-ray/CT, and multi-sensor fusion)
**Algorithms (Q2)**	Approach family (traditional ML, deep learning, classical computer vision, hybrid), task formulation (classification/detection/segmentation/regression), and training (supervised/unsupervised)
**Deployment (Q2)**	Local, edge, cloud, hybrid, or not reported processing; real-time constraints; and reported compute/hardware information
**Performance and Feasibility (Q2)**	Reported metrics (e.g., accuracy, precision, recall, F1, mAP, AUC, error rate), latency when available, and deployment constraints (lighting, dust, noise, occlusion, speed)
**Advanced Modeling Techniques (Q3)**	Presence or absence of remaining useful life/prognosis modeling, physics-informed modeling, principled multi-sensor fusion (versus feature-level concatenation), domain adaptation or transfer learning for cross-site generalization, calibrated uncertainty quantification, post-hoc explainability (e.g., class activation mapping), and digital-twin integration
**Evidence Gaps (Q3)**	Dataset availability, labeling strategy, reproducibility artifacts (code/data), external validation, statistical reporting (variance, repeated runs, confidence intervals), and cost reporting.

**Table 2:** Conveyor system anomalies, principal mechanisms, and representative included studies.

Component	Anomaly/Target	Typical Mechanisms or Causes	References
**Belt**	Longitudinal tears/rips	Foreign object penetration, sharp impacts, progressive fatigue, and local stress concentration.	[15,18,50,51]
	Surface damage (cracks, scratches, wear, breakage)	Abrasive loading, repeated friction, impact by conveyed material, aging, and environmental exposure.	[5,35,52,53,54]
	Deviation/misalignment	Off-center loading, uneven belt tension, skewed or damaged rollers, and geometric misalignment.	[55,56,57,58]
	Steel cord defects (breakage, corrosion, internal damage)	Cyclic fatigue, corrosion, manufacturing defects, local stress concentration, and progressive internal damage.	[59,60,61,62,63]
	Splice degradation/failure	Poor splice condition, local material degradation, repeated tensile loading, fatigue, and damage propagation.	[61,63,64]
**Idler/Roller**	Bearing and rotation faults	Lubrication loss, contamination, fatigue, wear, misalignment, and progressive rotational resistance.	[2,13,21,65]
	Overheating/thermal anomalies	Friction, blocked or slow-rotating idlers, bearing degradation, and localized mechanical resistance.	[16,17,66,67,68]
	Abnormal vibration	Imbalance, misalignment, bearing wear, looseness, contamination, and load-dependent dynamic changes.	[19,20,43,69,70]
	Acoustic anomalies	Internal friction, impact, loosened components, bearing degradation, and abnormal rotational dynamics.	[2,4,13,71,72]
**System**	Chute blockage/transfer-point obstruction	Oversized material, accumulated debris, foreign objects, irregular material flow, and transfer point congestion.	[26]
	Foreign object intrusion	Mining debris, anchor rods, steel pieces, gangue, wood, and operational spillage entering the belt path.	[73,74,75,76]
	Motor/drive-system anomalies	Mechanical or electrical degradation, imbalance, misalignment, load variation, looseness, and abnormal operating states.	[1,9,30,58]
	Load and operational performance anomalies	Overloading, speed variation, unstable operating regimes, sensor drift, and inefficient system operation.	[3,24,27,45,77]

**Table 3:** Scientific and technical research gaps in intelligent conveyor belt fault detection.

Research Gap	Current State of the Evidence	Consequence for the Field	Required Development Direction
Diagnosis-to-prognosis disconnect	RUL estimated in 3/131 studies (2.3%) and degradation trends forecast in 7/131 (5.3%); the remaining studies detect an already present fault	Detects that a fault exists, not when intervention is needed	RUL/degradation trajectory models built on existing detection outputs (e.g., survival analysis, trend extrapolation)
Absence of physics-informed modeling	No study uses physics-informed ML (0/131); 18/131 (13.7%) include a physics-based or NDT component, such as dynamic or bearing-life models, without embedding it in the learning model	Detectors may not extrapolate beyond their training distribution	Hybrid architectures coupling learned detectors with belt/cord/rubber degradation mechanics
Fusion without a fusion architecture	27/131 studies (20.6%) use several sensors, but only 3/131 (2.3%) apply a principled fusion architecture; the rest rely on feature concatenation or rule-based combination	Unclear whether reported gains reflect genuine complementary information or just added features	Principled fusion (attention-based or Bayesian) with explicit per-modality weighting
No cross-site transfer technique	No study applies explicit cross-site domain adaptation (0/131); 18/131 (13.7%) use generic transfer learning or pretraining, and one adapts from handheld to drone recordings of the same installation	The transferability gap (Section 3.3) has no corresponding technical solution in progress	Domain adaptation methods designed to close source-to-target performance gaps
No calibrated uncertainty in outputs	Predictive uncertainty quantified in 1/131 studies (0.8%), without demonstrated calibration; elsewhere “Bayesian” appears mainly as a training or optimization method, not as uncertainty quantification	Model outputs carry no confidence measure, blocking automated decision-making	Calibrated uncertainty (ensembles, conformal prediction) to flag low confidence cases
Limited model transparency	Post hoc explainability applied in 6/131 studies (4.6%)	Operators cannot verify why a model flagged an anomaly, limiting trust	Routine explainability reporting (Grad-CAM, SHAP) for safety-relevant faults
Isolated diagnostics, no system-level integration	Digital twin integrated in 3/131 studies (2.3%), one of them driven by simulated fault data; the rest evaluate detection in isolation	Detection is disconnected from plant-level decision-making	Closer integration with digital-twin or system-level operational models

## Data Availability

The study-level data extraction dataset supporting the findings of this review is available from the corresponding author upon reasonable request.

## Supplementary Materials

The following supporting information can be downloaded at https://www.mdpi.com/article/10.3390/s26216741/s1; Table S1: Database-specific search implementation and search yields. The four data-source yields sum to the 1566 records reported in the PRISMA flow diagram (Figure 1). The final search was completed in September 2026. The three-group Boolean query reported in Section 2.1 was adapted to the search fields available in each source; the MDPI platform and Google Scholar required simplified combinations, as described below; Table S2: PRISMA 2020 Checklist; Table S3: Study-level methodological concerns ratings (adapted JBI checklist; *n* = 131). Ratings: Y = Yes; P = Partly; N = No; U = Unclear. Domains: CO = Clear objective; SD = Study design; DD = Dataset description; GT = Ground truth; MD = Methodology detail; EP = Evaluation protocol; BC = Baseline comparison; SA = Statistical analysis; EV = External validation; RP = Reproducibility; Table S4: Sensing modalities in the 131 included studies: frequency, analytical approach, fault targets, and practical strengths and limitations.

## Abbreviations

The following abbreviations are used in this manuscript:

PRISMA	Preferred Reporting Items for Systematic Reviews and Meta-Analyses
JBI	Joanna Briggs Institute
CNN	Convolutional Neural Network
YOLO	You Only Look Once
ResNet	Residual Network
U-Net	U-Shaped Network
LSTM	Long Short-Term Memory
GRU	Gated Recurrent Unit
AI	Artificial Intelligence
IIoT	Industrial Internet of Things
ML	Machine Learning
NDT	Non-Destructive Testing
IoT	Internet of Things
RF	Random Forest
SVM	Support Vector Machine
DL	Deep Learning
VGG	Visual Geometry Group
RNN	Recurrent Neural Network
CV	Computer Vision
ROI	Region of Interest
UAV	Unmanned Aerial Vehicle
UGV	Unmanned Ground Vehicle
GPU	Graphics Processing Unit
PLCs	Programmable Logic Controllers
MCU	Microcontroller Unit
RUL	Remaining Useful Life
