# Supplementary material for: From Detection to Prediction: A Systematic Review of Intelligent Fault Detection in Industrial Conveyor Belt Systems
Source: Sensors (Basel). 2026 Oct 24;26(21):6741. doi: 10.3390/s26216741 (PMC13765607; doi:10.3390/s26216741)
Supplement: Supplementary file 1 [file sensors-26-06741-s001.zip › sensors-4577030-supplementary.pdf]

Supplementary materials

Table S1. Database-specific search implementation and search yields. The four data-source yields sum to the 1566 records reported in the PRISMA flow diagram (Figure 1). The final search was completed in September 2026. The three-group Boolean query reported in Section 2.1 was adapted to the search fields available in each source; the MDPI platform and Google Scholar required simplified combinations, as described below.

Source	Search field(s)	Search implementation	Limits	Records
Scopus	TITLE-ABS-KEY	Three-group Boolean query from Section 2.1 (conveyor terms AND problem-type terms AND [task terms OR method terms]), entered in TITLE-ABS-KEY and adapted to the platform syntax.	English; 2010–2026; journal articles	1010
IEEE Xplore	All Metadata	Three-group Boolean query from Section 2.1 (conveyor terms AND problem-type terms AND [task terms OR method terms]), entered in All Metadata and adapted to the platform syntax.	English; 2010–2026; journal articles	128
MDPI platform	All fields	Simplified combinations of conveyor, problem-type, and task or method terms; additional searches using "idler" and "steel cord" were run to identify component-specific studies. Sensing modality was assessed during screening rather than imposed as a search term.	English; 2010–2026; journal articles	77
Google Scholar (Publish or Perish)	Title words / keywords	Three-group Boolean query from Section 2.1 (conveyor terms AND problem-type terms AND [task terms OR method terms]); results were exported and deduplicated with the database records.	English; 2010–2026	351
Total				1566

Table S2. PRISMA 2020 Checklist

Section and Topic	Item	Checklist item	Location where item is reported
**TITLE**			
Title	1	Identify the report as a systematic review.	Title.
**ABSTRACT**			
Abstract	2	See the PRISMA 2020 for Abstracts checklist.	Abstract.
**INTRODUCTION**			
Rationale	3	Describe the rationale for the review in the context of existing knowledge.	Section 1.
Objectives	4	Provide an explicit statement of the objective(s) or question(s) the review addresses.	Section 2.1.
**METHODS**			
Eligibility criteria	5	Specify the inclusion and exclusion criteria for the review and how studies were grouped for the syntheses.	Section 2.1.
Information sources	6	Specify all databases, registers, websites, organisations, reference lists and other sources searched or consulted. Specify the date when each source was last searched or consulted.	Section 2.1; Table S1. Final search completed September 2026.
Search strategy	7	Present the full search strategies for all databases, registers and websites, including any filters and limits used.	Section 2.1; Table S1.

Section and Topic	Item	Checklist item	Location where item is reported
Selection process	8	Specify the methods used to decide whether a study met the inclusion criteria, including how many reviewers screened each record/report, whether independently, and any automation tools used.	Section 2.1.
Data collection process	9	Specify the methods used to collect data from reports, including how many reviewers, whether independently, and any automation tools used.	Section 2.1 (independent extraction by two authors).
Data items	10a	List and define all outcomes for which data were sought, and how results compatible with each outcome domain were selected.	Table 1.
	10b	List and define all other variables for which data were sought, and describe assumptions about missing/unclear information.	Table 1.
Study methodological-concern assessment	11	Specify the methods used to assess methodological-concern, including the tool(s), how many reviewers, whether independently, and automation tools used.	Section 2.2; Table S3.
Effect measures	12	Specify for each outcome the effect measure(s) used in the synthesis or presentation of results.	Not applicable. No meta-analysis was conducted.
Synthesis methods	13a	Describe the processes used to decide which studies were eligible for each synthesis.	Table 1; Section 4.
	13b	Describe methods to prepare data for presentation/synthesis (handling missing summary statistics, data conversions).	Section 2.1 (primary-category and outcome-selection rules).
	13c	Describe methods used to tabulate or visually display results of individual studies and syntheses.	Figures 2–6; Tables 1–3.
	13d	Describe methods used to synthesize results and the rationale. If meta-analysis, describe model(s) and software.	Sections 3–4. No meta-analysis. Python used for the descriptive statistics and figures.
	13e	Describe methods used to explore possible causes of heterogeneity among study results.	Not applicable. No meta-analysis was conducted.
	13f	Describe any sensitivity analyses conducted.	Not applicable. No meta-analysis was conducted.
Reporting bias assessment	14	Describe any methods used to assess risk of bias due to missing results in a synthesis.	Not applicable. Not conducted.
Certainty assessment	15	Describe any methods used to assess certainty (confidence) in the body of evidence.	Not applicable. Not conducted.
**RESULTS**			
Study selection	16a	Describe the results of the search and selection process, ideally using a flow diagram.	Figure 1; Section 3.1.
	16b	Cite studies that might appear to meet the inclusion criteria but were excluded, and explain why.	Section 3.1; Figure 1
Study characteristics	17	Cite each included study and present its characteristics.	Section 3.1; Table 2.
methodological concern in studies	18	Present assessments of methodological concern for each included study.	Figure 3; Table S3.

Section and Topic	Item	Checklist item	Location where item is reported
Results of individual studies	19	For all outcomes, present summary statistics and/or effect estimates for each study, ideally using structured tables or plots.	Section 4; Figure 5D.
Results of syntheses	20a	For each synthesis, briefly summarise the characteristics and methodological concern among contributing studies.	Sections 3–4.
	20b	Present results of all statistical syntheses (meta-analysis), including summary estimates and heterogeneity.	Not applicable. No meta-analysis was conducted.
	20c	Present results of all investigations of possible causes of heterogeneity.	Not applicable. No meta-analysis was conducted.
	20d	Present results of all sensitivity analyses.	Not applicable. No meta-analysis was conducted.
Reporting biases	21	Present assessments of risk of bias due to missing results for each synthesis assessed.	Not applicable. Not conducted.
Certainty of evidence	22	Present assessments of certainty (confidence) in the body of evidence for each outcome assessed.	Not applicable. Not conducted.
**DISCUSSION**			
Discussion	23a	Provide a general interpretation of the results in the context of other evidence.	Section 5; Section 6.
	23b	Discuss any limitations of the evidence included in the review.	Section 5.1.
	23c	Discuss any limitations of the review processes used.	Section 5.2 (limitations paragraph),
	23d	Discuss implications of the results for practice, policy, and future research.	Section 5.2; Section 6.
**OTHER INFORMATION**			
Registration and protocol	24a	Provide registration information, including register name and number, or state that the review was not registered.	Section 2.1. Not registered.
	24b	Indicate where the review protocol can be accessed, or state that a protocol was not prepared.	Section 2.1.
	24c	Describe and explain any amendments to information provided at registration or in the protocol.	Section 5.2 (limitations paragraph): the search strategy was refined and rerun after an initial analysis; eligibility criteria and appraisal rules were unchanged.
Support	25	Describe sources of financial or non-financial support for the review, and the role of funders/sponsors.	Funding statement.
Competing interests	26	Declare any competing interests of review authors.	Conflicts of Interest statement.
Availability of data, code and other materials	27	Report which of the following are publicly available and where they can be found: template data collection forms; extracted data; data used for all analyses; analytic code; other materials used in the review.	Data Availability Statement; Tables S3 and extraction dataset available on request.

From: Page MJ, McKenzie JE, Bossuyt PM, Boutron I, Hoffmann TC, Mulrow CD, et al. The PRISMA 2020 statement: an updated guideline for reporting systematic reviews. BMJ 2021;372:n71.

Table S3. Study-level methodological concerns ratings (adapted JBI checklist; n = 131). Ratings: Y = Yes; P = Partly; N = No; U = Unclear. Domains: CO = Clear objective; SD = Study design; DD = Dataset description; GT = Ground truth; MD = Methodology detail; EP = Evaluation protocol; BC = Baseline comparison; SA = Statistical analysis; EV = External validation; RP = Reproducibility.

Study ID	CO	SD	DD	GT	MD	EP	BC	SA	EV	RP	Y	P	N/U	Score	Overall
1	Y	Y	Y	P	Y	Y	Y	P	N	N	6	2	2	70.0%	MODERATE
2	Y	Y	U	P	P	U	P	N	N	N	2	3	5	35.0%	HIGH
3	Y	Y	P	P	Y	P	P	N	N	N	3	4	3	50.0%	HIGH
4	Y	Y	U	P	Y	P	P	N	N	N	3	3	4	45.0%	HIGH
5	Y	Y	Y	P	Y	Y	Y	N	N	N	6	1	3	65.0%	MODERATE
6	Y	Y	Y	P	Y	P	Y	N	N	N	5	2	3	60.0%	MODERATE
7	Y	Y	P	P	Y	P	Y	P	N	N	4	4	2	60.0%	MODERATE
8	Y	Y	Y	Y	Y	Y	Y	P	U	N	7	1	2	75.0%	MODERATE
9	Y	Y	Y	Y	Y	Y	Y	Y	Y	P	9	1	0	95.0%	LOW
10	Y	Y	P	Y	Y	N	N	N	N	N	4	1	5	45.0%	HIGH
11	Y	Y	Y	P	Y	Y	Y	N	N	N	6	1	3	65.0%	MODERATE
12	Y	Y	Y	Y	Y	P	Y	N	P	P	6	3	1	75.0%	MODERATE
13	Y	Y	P	Y	Y	P	Y	N	U	N	5	2	3	60.0%	MODERATE
14	Y	Y	Y	P	P	N	N	N	N	N	3	2	5	40.0%	HIGH
15	Y	Y	Y	Y	P	Y	Y	P	N	P	6	3	1	75.0%	MODERATE
16	Y	Y	Y	P	Y	Y	Y	N	U	N	6	1	3	65.0%	MODERATE
17	Y	Y	Y	P	Y	N	N	N	N	N	4	1	5	45.0%	HIGH
18	Y	Y	Y	Y	Y	Y	Y	P	N	N	7	1	2	75.0%	MODERATE
19	Y	Y	P	Y	Y	P	Y	N	N	P	5	3	2	65.0%	MODERATE
20	Y	Y	Y	P	Y	P	Y	N	U	N	5	2	3	60.0%	MODERATE
21	Y	Y	Y	P	Y	Y	Y	N	Y	P	7	2	1	80.0%	MODERATE
22	Y	Y	P	P	Y	Y	Y	N	U	N	5	2	3	60.0%	MODERATE
23	Y	Y	Y	P	Y	Y	Y	N	P	N	6	2	2	70.0%	MODERATE
24	Y	Y	Y	P	P	P	Y	N	P	N	4	4	2	60.0%	MODERATE
25	Y	Y	Y	P	Y	Y	Y	N	U	N	6	1	3	65.0%	MODERATE
26	Y	Y	Y	P	Y	Y	Y	N	N	N	6	1	3	65.0%	MODERATE
27	Y	Y	P	Y	Y	Y	N	P	N	P	5	3	2	65.0%	MODERATE
28	Y	Y	Y	P	Y	P	Y	N	N	N	5	2	3	60.0%	MODERATE
29	Y	Y	P	P	Y	Y	Y	P	U	P	5	4	1	70.0%	MODERATE
30	Y	Y	Y	P	Y	Y	Y	N	U	N	6	1	3	65.0%	MODERATE
31	Y	Y	Y	P	Y	P	Y	Y	Y	P	7	3	0	85.0%	MODERATE
32	Y	Y	Y	P	Y	Y	Y	N	U	N	6	1	3	65.0%	MODERATE
33	Y	Y	Y	P	Y	Y	Y	N	N	P	6	2	2	70.0%	MODERATE
34	Y	Y	Y	P	Y	Y	Y	P	N	N	6	2	2	70.0%	MODERATE
35	Y	Y	Y	P	Y	Y	Y	N	N	P	6	2	2	70.0%	MODERATE
36	Y	Y	P	P	Y	Y	Y	N	U	P	5	3	2	65.0%	MODERATE
37	Y	Y	P	P	Y	P	Y	N	N	P	4	4	2	60.0%	MODERATE
38	Y	Y	Y	P	Y	Y	Y	P	N	N	6	2	2	70.0%	MODERATE
39	Y	Y	P	P	Y	Y	Y	P	Y	N	6	3	1	75.0%	MODERATE
40	Y	Y	Y	P	Y	Y	Y	N	U	N	6	1	3	65.0%	MODERATE
41	Y	Y	Y	P	Y	Y	N	P	N	N	5	2	3	60.0%	MODERATE
42	Y	Y	Y	P	Y	Y	Y	Y	N	P	7	2	1	80.0%	MODERATE
43	Y	Y	Y	P	Y	Y	Y	N	N	P	6	2	2	70.0%	MODERATE
44	Y	Y	P	P	Y	Y	Y	N	U	P	5	3	2	65.0%	MODERATE
45	Y	Y	P	P	Y	Y	Y	N	N	N	5	2	3	60.0%	MODERATE
46	Y	Y	Y	P	Y	Y	Y	N	N	N	6	1	3	65.0%	MODERATE
47	Y	Y	Y	P	Y	Y	Y	N	Y	N	7	1	2	75.0%	MODERATE
48	Y	Y	Y	P	Y	P	Y	N	U	N	5	2	3	60.0%	MODERATE
49	Y	Y	Y	P	Y	Y	Y	N	Y	N	7	1	2	75.0%	MODERATE
50	Y	Y	P	U	Y	P	Y	N	U	N	4	2	4	50.0%	HIGH
51	Y	Y	Y	P	Y	Y	Y	N	N	N	6	1	3	65.0%	MODERATE
52	Y	Y	Y	P	Y	Y	Y	N	N	Y	7	1	2	75.0%	MODERATE
53	Y	Y	P	Y	Y	Y	N	N	U	N	5	1	4	55.0%	HIGH
54	Y	Y	Y	P	Y	Y	Y	N	N	Y	7	1	2	75.0%	MODERATE
55	Y	Y	Y	Y	Y	P	Y	N	U	P	6	2	2	70.0%	MODERATE
56	Y	Y	Y	Y	Y	Y	Y	N	U	N	7	0	3	70.0%	MODERATE
57	Y	Y	U	P	Y	P	Y	N	N	N	4	2	4	50.0%	HIGH

Study ID	CO	SD	DD	GT	MD	EP	BC	SA	EV	RP	Y	P	N/U	Score	Overall
58	Y	Y	Y	P	Y	Y	Y	N	U	N	6	1	3	65.0%	MODERATE
59	Y	Y	Y	P	Y	P	Y	N	N	P	5	3	2	65.0%	MODERATE
60	Y	Y	Y	P	Y	Y	Y	N	U	P	6	2	2	70.0%	MODERATE
61	Y	P	Y	U	Y	Y	Y	P	N	N	5	2	3	60.0%	MODERATE
62	Y	Y	P	P	Y	P	Y	N	N	N	4	3	3	55.0%	HIGH
63	Y	Y	Y	P	Y	Y	Y	P	N	P	6	3	1	75.0%	MODERATE
64	Y	Y	P	P	Y	P	N	N	N	N	3	3	4	45.0%	HIGH
65	Y	Y	Y	P	Y	Y	Y	N	U	P	6	2	2	70.0%	MODERATE
66	Y	Y	P	P	Y	Y	Y	N	N	N	5	2	3	60.0%	MODERATE
67	Y	Y	Y	P	Y	Y	Y	N	N	N	6	1	3	65.0%	MODERATE
68	Y	Y	Y	P	Y	Y	Y	N	N	P	6	2	2	70.0%	MODERATE
69	Y	Y	Y	P	Y	Y	Y	N	Y	N	7	1	2	75.0%	MODERATE
70	Y	Y	Y	P	Y	Y	Y	N	Y	N	7	1	2	75.0%	MODERATE
71	Y	Y	Y	P	Y	Y	Y	N	N	N	6	1	3	65.0%	MODERATE
72	Y	Y	P	P	Y	Y	Y	P	N	P	5	4	1	70.0%	MODERATE
73	Y	Y	Y	P	Y	Y	Y	N	Y	P	7	2	1	80.0%	MODERATE
74	Y	Y	P	N	Y	Y	Y	P	P	N	5	3	2	65.0%	HIGH
75	Y	Y	P	P	P	Y	Y	N	N	N	4	3	3	55.0%	HIGH
76	Y	Y	Y	P	Y	Y	Y	Y	N	P	7	2	1	80.0%	MODERATE
77	Y	P	P	P	Y	Y	Y	Y	N	P	5	4	1	70.0%	MODERATE
78	Y	Y	Y	Y	Y	Y	Y	Y	P	N	8	1	1	85.0%	LOW
79	Y	Y	Y	P	Y	Y	Y	N	N	P	6	2	2	70.0%	MODERATE
80	Y	Y	Y	P	Y	Y	Y	N	N	N	6	1	3	65.0%	MODERATE
81	Y	P	P	P	Y	Y	P	N	P	P	3	6	1	60.0%	MODERATE
82	Y	Y	Y	Y	Y	Y	Y	N	P	P	7	2	1	80.0%	LOW
83	Y	Y	P	Y	Y	Y	Y	P	N	N	6	2	2	70.0%	MODERATE
84	Y	Y	Y	P	Y	Y	Y	N	N	P	6	2	2	70.0%	MODERATE
85	Y	Y	Y	Y	Y	Y	Y	N	P	P	7	2	1	80.0%	LOW
86	Y	Y	Y	Y	Y	Y	Y	N	N	N	7	0	3	70.0%	MODERATE
87	Y	Y	Y	Y	Y	Y	Y	Y	P	N	8	1	1	85.0%	LOW
88	Y	Y	Y	Y	Y	Y	Y	Y	P	N	8	1	1	85.0%	LOW
89	Y	Y	P	P	Y	Y	N	Y	P	N	5	3	2	65.0%	MODERATE
90	Y	Y	Y	Y	Y	Y	Y	N	P	N	7	1	2	75.0%	MODERATE
91	Y	Y	P	P	Y	Y	N	N	P	N	4	3	3	55.0%	HIGH
92	Y	Y	P	Y	Y	Y	N	N	N	N	5	1	4	55.0%	HIGH
93	Y	Y	Y	P	Y	Y	Y	Y	Y	P	8	2	0	90.0%	MODERATE
94	Y	Y	P	P	Y	Y	Y	Y	N	N	6	2	2	70.0%	MODERATE
95	Y	Y	P	P	Y	P	Y	N	P	N	4	4	2	60.0%	MODERATE
96	Y	Y	P	P	Y	Y	P	N	N	P	4	4	2	60.0%	MODERATE
97	Y	Y	P	P	Y	Y	Y	N	N	N	5	2	3	60.0%	MODERATE
98	Y	Y	Y	Y	Y	Y	Y	Y	N	P	8	1	1	85.0%	LOW
99	Y	Y	P	P	Y	Y	Y	N	N	N	5	2	3	60.0%	MODERATE
100	Y	Y	Y	P	Y	Y	Y	N	P	P	6	3	1	75.0%	MODERATE
101	Y	Y	P	Y	Y	P	Y	N	N	N	5	2	3	60.0%	MODERATE
102	Y	Y	Y	Y	Y	Y	Y	P	N	P	7	2	1	80.0%	LOW
103	Y	Y	Y	P	Y	Y	Y	N	P	P	6	3	1	75.0%	MODERATE
104	Y	Y	Y	P	Y	Y	Y	P	P	N	6	3	1	75.0%	MODERATE
105	Y	Y	Y	P	Y	Y	Y	N	P	N	6	2	2	70.0%	MODERATE
106	Y	Y	P	Y	Y	Y	Y	Y	N	P	7	2	1	80.0%	MODERATE
107	Y	Y	P	P	Y	Y	N	N	P	N	4	3	3	55.0%	HIGH
108	Y	Y	Y	Y	Y	Y	N	N	P	P	6	2	2	70.0%	MODERATE
109	Y	Y	P	Y	Y	Y	Y	N	P	P	6	3	1	75.0%	MODERATE
110	Y	Y	P	Y	Y	Y	Y	Y	P	N	7	2	1	80.0%	MODERATE
111	Y	Y	Y	P	Y	Y	Y	N	P	P	6	3	1	75.0%	MODERATE
112	Y	Y	Y	Y	Y	Y	Y	P	N	N	7	1	2	75.0%	MODERATE
113	Y	Y	P	Y	Y	Y	N	Y	N	N	6	1	3	65.0%	MODERATE
114	Y	Y	P	Y	Y	Y	P	N	N	N	5	2	3	60.0%	MODERATE
115	Y	Y	P	P	Y	Y	Y	Y	N	N	6	2	2	70.0%	MODERATE
116	Y	Y	P	Y	Y	Y	P	N	N	N	5	2	3	60.0%	MODERATE
117	Y	Y	P	P	Y	Y	Y	N	N	N	5	2	3	60.0%	MODERATE
118	Y	Y	P	Y	Y	Y	Y	N	N	N	6	1	3	65.0%	MODERATE
119	Y	Y	P	P	Y	Y	N	N	N	N	4	2	4	50.0%	HIGH
120	Y	Y	Y	P	Y	Y	P	N	N	P	5	3	2	65.0%	MODERATE

Study ID	CO	SD	DD	GT	MD	EP	BC	SA	EV	RP	Y	P	N/U	Score	Overall
121	Y	Y	Y	Y	Y	Y	N	N	N	N	6	0	4	60.0%	MODERATE
122	Y	Y	Y	P	Y	Y	Y	N	Y	P	7	2	1	80.0%	MODERATE
123	Y	Y	Y	P	P	P	N	N	N	N	3	3	4	45.0%	HIGH
124	Y	P	N	P	Y	N	N	N	N	N	2	2	6	30.0%	HIGH
125	Y	Y	Y	P	Y	Y	Y	N	P	N	6	2	2	70.0%	MODERATE
126	Y	Y	P	P	Y	P	Y	N	N	N	4	3	3	55.0%	HIGH
127	Y	Y	P	Y	Y	P	N	N	N	N	4	2	4	50.0%	HIGH
128	Y	Y	P	P	P	P	N	P	N	N	2	5	3	45.0%	HIGH
129	Y	Y	P	P	Y	P	Y	N	P	U	4	4	2	60.0%	MODERATE
130	Y	Y	P	P	Y	P	Y	N	P	N	4	4	2	60.0%	MODERATE
131	Y	Y	P	P	Y	P	Y	N	N	U	4	3	3	55.0%	HIGH

Table S4. Sensing modalities in the 131 included studies: frequency, analytical approach, fault targets, and practical strengths and limitations.

Sensing modality (data acquired)	Studies, n (%)	Real-time, n	Primary analytical category, n	Main fault targets, n	Main strengths	Main limitations	Representative studies
**Vision / camera** RGB and line-scan images, video, laser or structured-light profiles, 3D point clouds	53 (40.5)	44	DL 40; CV 9; ML 4	Belt surface damage 29; deviation 16; foreign objects 15	Dense spatial information at moderate hardware cost; supports detection, localization, and geometric measurement. Laser and structured-light set-ups add depth for tear profiles and small deformations.	Sensitive to illumination, dust, occlusion, motion blur, and camera geometry; observes only the visible belt surface. Only 5 of 53 studies used data from more than one site.	[5,6,18,52,55]
**Multi-sensor fusion** Combinations of vibration, temperature, acoustic, current, load, speed, image, or LiDAR data, often from IoT or robot-mounted nodes	27 (20.6)	13	ML 9; DL 9; CV 4; PB 2; SP/NDT 3	Drive/mechanical 20; idler/roller 15; thermal 9	Complementary channels can compensate when one signal is weak; suited to continuous, system-level monitoring with local preprocessing.	Synchronization, calibration, power, bandwidth, and maintenance add complexity. Only 3 of 27 studies used learned or adaptive fusion; the others aggregated features, applied rules, or used the channels separately.	[27,66,72,98,131]
**Vibration** Acceleration or strain time series from contact accelerometers, belt-mounted sensors, or fibre accelerometers	16 (12.2)	5	ML 9; DL 6; SP/NDT 1	Idler/roller 15; drive/mechanical 13	Sensitive to bearing and roller dynamics; mature time–frequency feature extraction; low data rates suit edge processing.	Needs reliable mounting and many measurement points on long lines; signals change with load, speed, and transmission paths. 11 of 16 studies were laboratory-based.	[19–21,65,69]
**Acoustic** Airborne sound from fixed microphones or handheld and robot-mounted recorders	13 (9.9)	4	DL 8; ML 3; SP/NDT 2	Idler/roller 12; drive/mechanical 9	Non-contact; one microphone can cover several idlers and suits mobile inspection routes. Field recordings were used in 10 of 13 studies.	Affected by background noise from nearby equipment, microphone placement, and reverberation; acoustic signatures vary between sites.	[2,4,13,71,113]
**Thermal infrared** Radiometric images or temperature fields from fixed, robot-mounted, or UAV cameras	7 (5.3)	3	DL 4; CV 3	Thermal anomalies 6; idler/roller 5	Non-contact localization of hot spots; detects overheated idlers and friction heating associated with longitudinal tears.	Affected by emissivity, viewing angle, ambient temperature, and dust; detection performance can depend on conveyor load and transport rate.	[17,50,68,79,123]

**Sensing modality (data acquired)**	**Studies, n (%)**	**Real-time, n**	**Primary analytical category, n**	**Main fault targets, n**	**Main strengths**	**Main limitations**	**Representative studies**
**Magnetic / electromagnetic NDT** Magnetic flux, weak-magnetic, or inductive signals across the belt width	7 (5.3)	0	ML 2; SP/NDT 3; DL 2	Steel-cord defects 6; belt surface damage 3	Detects internal cord breaks, corrosion, and splice defects that are not visible at the belt surface.	Requires specialized hardware and calibration; results depend on sensor position, thresholds, and the defect types represented in the data. No study reported real-time operation, and 5 of 7 were laboratory-based.	[59,60,62–64]
**Electrical** Motor current, power, or energy-consumption signals from the drive	3 (2.3)	2	ML 2; SP/NDT 1	Drive/mechanical 2; deviation 1; belt tension 1	Uses signals already available at the drive, so no sensors are needed along the belt line.	Indirect for localized belt or idler defects; signatures shift with load and control actions. All three studies were laboratory or simulation based.	[30,58,77]
**Distributed optical fibre** Distributed vibration signals along a fibre (Φ-OTDR distributed acoustic sensing)	2 (1.5)	2	DL 2	Idler/roller 2	A single fibre can cover the full line length and locate faulty idlers; passive and immune to electromagnetic interference.	Interrogator cost, coupling of the fibre to the structure, and large data volumes. Four further fibre-based studies (DAS or FBG accelerometers) are coded under vibration.	[95,96]
**Pressure / force** Force or pressure signals from contact sensors	2 (1.5)	1	ML 1; PB 1	Belt surface damage 2; drive/mechanical 1	Direct measurement of the mechanical response of the belt to damage.	Contact sensing; evidence limited to two laboratory studies.	[11,12]
**Maintenance records** Repair, failure, and operating-condition records kept by the mine (no sensor data)	1 (0.8)	0	DL 1	Belt surface damage 1; steel-cord defects 1	Uses records that mines already keep and supports forecasting of belt deterioration over its service life.	Depends on the completeness of manual inspection and repair records; no direct measurement of belt condition.	[138]
